# White and brown adipose tissue share a convergent fibro-adipogenic progenitor population

**DOI:** 10.1038/s44319-025-00591-6

**Published:** 2025-10-08

**Authors:** Hoang V Bui, Julia K Hansen, Valentina Lo Sardo, Andrea Galmozzi

**Affiliations:** 1 Department of Medicine, University of Wisconsin-Madison, School of Medicine and Public Health, Madison, WI USA; 2 Nutrition and Metabolism Graduate Program, University of Wisconsin-Madison, Madison, WI USA; 3 Department of Cell and Regenerative Biology, University of Wisconsin-Madison, School of Medicine and Public Health, Madison, WI USA; 4 University of Wisconsin Carbone Cancer Center, University of Wisconsin-Madison School of Medicine and Public Health, Madison, WI USA; 5 Department of Biomolecular Chemistry, University of Wisconsin-Madison School of Medicine and Public Health, Madison, WI USA

**Keywords:** Adipose Tissue Heterogeneity, Committed Adipocyte Precursors, Fibro-adipogenic Progenitors, Singel-cell RNAseq, Adipose Tissue Development, Metabolism, Methods & Resources

## Abstract

Adipose tissue heterogeneity has emerged as a central factor in regulating adipose tissue function in physiology and pathophysiology, yet tools to model and study this diversity in vitro remain limited. Here, we performed single-cell RNA sequencing on cultured primary white and brown preadipocytes to assess how in vitro conditions impact progenitor identity. We identified two major subpopulations in both depots: committed adipogenic precursors (CAPs) and fibro-adipogenic progenitor-like cells (FAPLs). Remarkably, FAPLs were also present in brown adipose tissue, expanding the known landscape of progenitor populations in this depot. Trajectory and regulon analyses revealed that both white and brown FAPLs exhibit similar pro-fibrotic, stress-responsive signatures and diverge early from proliferating progenitor states. Integration of datasets showed that FAPLs from both depots cluster together, emphasizing their conserved identity, while CAPs remain depot-specific. Comparison to previously published in vivo single-cell datasets revealed that these in vitro populations, including brown adipose FAPLs, correspond to adipose-resident progenitor subtypes, validating the physiological relevance of this model for studying adipose tissue heterogeneity and development.

## Synopsis

Cultured neonatal white and brown preadipocytes recapitulate in vivo heterogeneity, defining two conserved lineages of committed adipogenic (CAPs) and fibro-adipogenic (FAPs) progenitors, and reveal a previously unrecognized fibro-adipogenic population in brown adipose tissue.

- Single-cell RNA sequencing of cultured neonatal white and brown preadipocytes revealed two conserved progenitor lineages: committed adipogenic precursors (CAPs) and fibro-adipogenic progenitor-like cells (FAPLs).
- Early progenitors followed bifurcated developmental trajectories, committing either to adipogenic (CAPs) or fibro-adipogenic (FAPLs) fates.
- Fibro-adipogenic progenitors are present in brown adipose tissue and are highly similar to their white adipose counterparts.
- White and brown FAPLs share a mesenchymal/fibro-adipogenic identity and display highly similar transcriptional signatures.
- In vitro APC populations align with known in vivo APC subtypes.

## Abstract (web summary)

Cultured neonatal white and brown preadipocytes recapitulate in vivo heterogeneity, defining two conserved lineages of committed adipogenic (CAPs) and fibro-adipogenic (FAPs) progenitors, and reveal a previously unrecognized fibro-adipogenic population in brown adipose tissue.

## Graphical abstract

**Figure:**
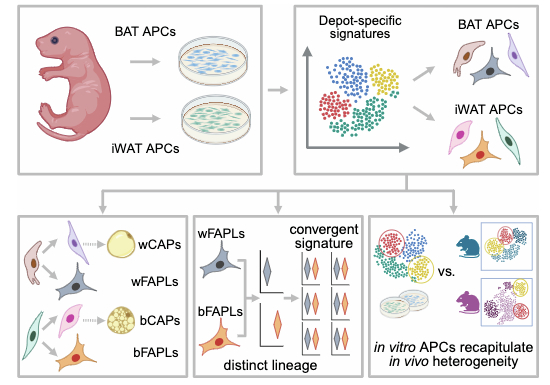

**Figure:**
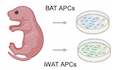

## Introduction

Beyond its fundamental role in storing and releasing energy, adipose depots substantially impact systemic physiology by means of endocrine signaling, regulation of inflammatory processes, and behavioral modulation (Chouchani and Kajimura, 2019; Ouchi et al, 2011; Stern et al, 2016). The adipose tissue is critical for systemic insulin sensitivity and glucose homeostasis, with impairments in adipocyte function tightly linked to the onset of obesity and type 2 diabetes (T2D) (Rosen and Spiegelman, 2006; Samuel et al, 2010). The two primary types of adipose tissue, white and brown adipose, exhibit distinct functional characteristics. White adipose tissue primarily stores energy in the form of fat, while brown adipose tissue actively participates in thermogenesis and energy expenditure (Pond, 1992). With new cutting-edge methodologies, it is now well-established that resident cell types like mature lipid-storing adipocytes, adipocyte precursor cells (APCs), and immune cells represent a heterogeneous population that collectively contributes to the overall functions and remodeling of fat depots in health and disease (Duerre and Galmozzi, 2022; Wang et al, 2022). Recent studies have further delineated the complexity within adipose tissues, identifying multiple subpopulations of adipocytes with distinct functions within both mouse and human adipose tissues. For example, LGAs/AdipoPLIN populations consist of insulin-responsive adipocytes involved in lipid synthesis, while LSAs/AdipoLEP populations rely more on lipid uptake rather than de novo synthesis (Backdahl et al, 2021; Sarvari et al, 2021). In interscapular brown adipose tissue (iBAT), two discrete subsets of brown adipocytes, high (BA-H) and low (BA-L) thermogenic adipocytes, respectively, have been identified based on the uneven expression of adiponectin and the thermogenic protein Ucp1 (Cinti et al, 2002; Song et al, 2020; Spaethling et al, 2016). Furthermore, a third class of mature brown adipocytes capable of regulating thermogenesis of neighboring cells by inhibiting thermogenic activity via production and secretion of acetate has been recently discovered in humans and mice (Sun et al, 2020; Sun et al, 2021).

Akin to fully mature fat cells, adipocyte progenitors (APCs) are a heterogeneous population that plays an integral part in the maintenance of healthy adipose tissue. Many studies applied single-cell RNAseq profiling in mice and humans at different ages and metabolic conditions (i.e., lean vs obese) to study the composition of WAT depots and led to the identification of as many APC subtypes. Fueled by slightly distinct cell sorting strategies, these APC clusters have been tagged with unique names, including adipogenesis-regulatory cells (Aregs) (Schwalie et al, 2018), Fibro-inflammatory Progenitors (FIPs) (Hepler et al, 2018), Fibroadipogenic Progenitors (FAPs) (Sarvari et al, 2021), or Dpp4^+^ multipotent progenitors (Burl et al, 2018; Merrick et al, 2019; Rondini et al, 2021). Despite this initial lack of consensus, recent efforts have tried to harmonize the nomenclature of white progenitor subtypes, resulting in two main classes consisting of preadipocytes, or Committed Adipocyte Progenitors (CAPs), and Fibro-Adipogenic Progenitors (FAPs) (Maniyadath et al, 2023). Conversely, the spectrum of brown adipocyte progenitors is less understood. One study identified several subtypes, primarily localized around the vasculature, including the adipogenic quiescent/cold-responsive ASC1 alongside less-defined ASC2 and ASC3 (Burl et al, 2018). However, whether these cells represent distinct progenitor subtypes or reflect different stages of maturation of the same brown adipocyte progenitors remains unclear (Karlina et al, 2021).

We recently optimized a method to isolate primary white and brown preadipocytes from neonatal mice (Galmozzi et al, 2021). Adipose depots of newborn animals are rapidly growing and are enriched in progenitor cells with high proliferative capacity and high differentiation potential (Wang et al, 2013). Here, we conducted single-cell RNAseq analysis in cultured primary white and brown preadipocytes to determine how accurately these in vitro models recapitulate the heterogeneity of adipocyte progenitors in vivo. Consistent with previous reports, we identified two main preadipocyte subpopulations for white preadipocytes, consisting of committed adipogenic progenitors (wCAPs) and fibroadipogenic progenitors (FAPs-like, or wFAPLs). Similarly, brown preadipocytes also display a bifurcated differentiation commitment, with one subpopulation of classical adipocyte precursors (bCAPs) and the other one presenting more fibro-inflammatory characteristics (bFAPLs). Notably, we show that wFAPLs and bFAPLs are remarkably similar and exhibit identical pro-fibrotic properties, suggesting a shared regulatory role in BAT and WAT function.

## Results

### Characterization of white and brown adipocyte precursors

**Figure 1:**
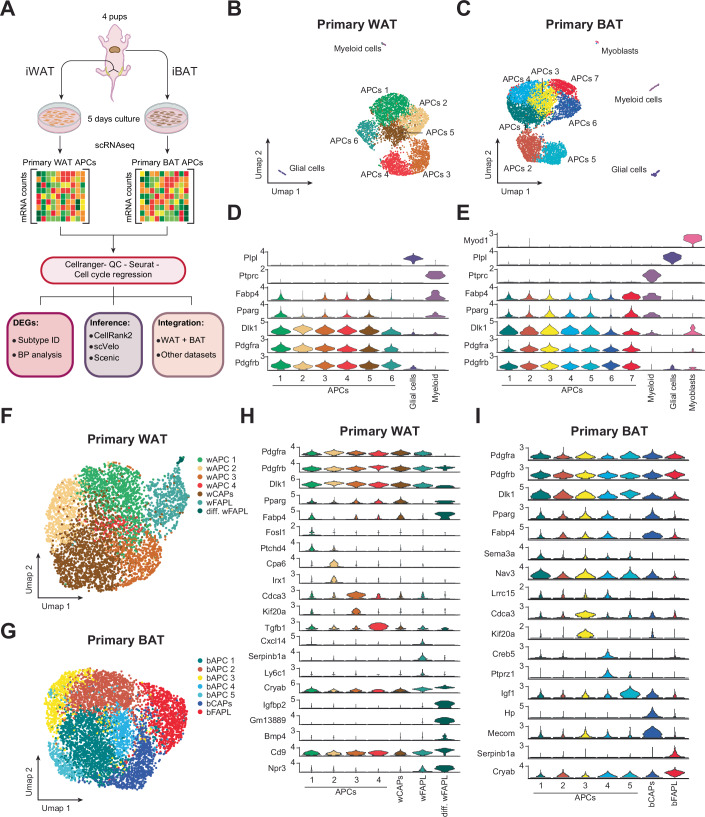
Cultured primary WAT and BAT preadipocytes maintain their heterogeneity. (**A**) Schematic of the single-cell RNA-seq pipeline used to isolate and characterize adipose progenitor cells (APCs) and downstream integrative analyses, including data quality control, cell cycle regression, and subpopulation annotation from neonatal white (WAT) and brown (BAT) adipose tissues. (**B**, **C**) UMAP embeddings showing global clustering of all recovered cell types, including APCs, myeloid cells, glial cells, and myoblasts in WAT (**B**) and BAT (**C**). (**D**, **E**) Expression of representative marker genes (e.g., *Pdgfra*, *Pdgfrb*, *Pparg*, *Dlk1*, *Fabp4*) highlights the identity of non-adipo cells in WAT (**D**) and BAT (**E**) (*n* = 4407 total cells for WAT and 6173 total cells for BAT). (**F**) UMAPs showing clusters of WAT APCs (*n* = 4331) after cell cycle regression. (**G**) UMAPs showing clusters of BAT APCs (*n* = 5958) after cell cycle regression. (**H**) Violin plots of representative markers of wAPC 1–4, wCAPs, wFAPLs, and differentiating wFAPLs (*n* = 4331 total cells). (**I**) Violin plots of representative markers of bAPC 1–5, bCAPs, and bFAPLs (*n* = 5958 total cells). Source data are available online for this figure.

Following isolation from a pool of four male and female C57/BL6 pups, white and brown adipose tissue progenitors were expanded in vitro for 5 days, trypsinized into a single-cell suspension and processed for single-cell RNA sequencing using the 10X Genomics platform (Fig. 1A). Unbiased clustering confirmed an enrichment of 98.28% and 97.08% for adipocyte precursor cells (APCs, *Pdgfra*^*+*^, *Pdgfrb*^*+*^, and *Dlk1*^*+*^) isolated from white and brown adipose depots, respectively (Fig. 1B–E). Non-adipose cells constituted a minor percentage of the total cell population in both WAT and BAT and were identified as myelin-producing glial cell (*Plp1*^*+*^, 1.07% in WAT and 1.11% in BAT), myeloid cells (*Ptprc*^*+*^, 0.66% in WAT and 1.27% in BAT) and myocytes (*Myod1*^*+*^, 0.54% in BAT) (Fig. 1B–E). Removal of non-adipose cells resulted in 4331 WAT and 5958 BAT progenitors that were used for downstream analysis (Fig. EV1A,F). To minimize the impact of cell cycle phase, which appeared to be a major determinant in defining cell populations (Fig. EV1B,C,G,H), we conducted cell cycle regression, re-clustered WAT (Fig. EV1D,E) and BAT progenitors (Fig. EV1I,J), and identified seven distinct clusters for both WAT (Fig. 1F) and BAT preadipocytes (Fig. 1G).

**Figure EV1:**

Cell cycle regression and re-clustering of WAT and BAT APCs. (**A**) Clustering of WAT APCs before cell cycle regression. (**B**) Cell cycle phase of WAT APCs. (**C**) Distribution of cell cycle in WAT APC clusters from (**A**). (**D**) Cell cycle regression leads to redistribution of WAT APCs. (**E**) Cell cycle phases are equally distributed across clusters upon cell cycle regression. (**F**) Clustering of BAT APCs before cell cycle regression. (**G**) Cell cycle phase of BAT APCs. (**H**) Distribution of cell cycle in BAT APC clusters from (**F**). (**I**) Cell cycle regression leads to redistribution of BAT APCs. (**J**) Cell cycle phases are equally distributed across BAT clusters upon cell cycle regression.

Like mesenchymal stromal cells (Gao et al, 2018; Turley et al, 2015; Vishvanath et al, 2016), all adipocyte progenitors from both WAT and BAT showed wide expression of platelet-derived growth factor receptors *Pdgfra* and *Pdgfrb* (Fig. 1H,I). Similarly, the common marker of preadipocytes *Dlk1* (Smas and Sul, 1993) was also widely expressed across all WAT and BAT clusters (Fig. 1H,I). Nevertheless, each WAT and BAT cluster was defined by a unique set of differentially expressed genes (Fig. EV2A,B; Datasets EV1 and EV2). Specifically, we identified four WAT clusters characterized by low expression of *Pparg* and a distinct gene signature (wAPC 1, *Fosl1*^*+*^ and *Ptchd4*^*+*^, 22.6%; wAPC 2, *Cpa6*^*+*^ and *Irx1*^*+*^, 14.8%; wAPC 3, *Cdca3*^*high*^ and *Kif20a*^+^*, 13*%; and wAPC 4, *Tgfb1*^*high*^, 2.9%) (Fig. 1H). Notably, one cluster showed significantly higher expression of canonical adipocyte markers (i.e., *Pparg*^*high*^, *Fabp4*^*high*^) and was therefore labeled as white adipocyte precursors, or wCAPs (Fig. 1F,H). Conversely, another cluster, that we called white Fibro-Adipogenic-Progenitor-Like (wFAPLs), was characterized by the expression of chemokines (*Cxcl14*^*+*^), stress response genes (*Cryab*^*high*^), and inflammation-related genes (*Ly6c1*^*+*^, *Serpinb1a*^*+*^), reminiscent of the FAPs previously identified in mouse and human WAT (Fig. 1F,H). Finally, a small percentage of these cells (*Igfbp2*^*+*^, *Gm13889*^*+*^*, Bmp4*^+^, *Cd9*^*high*^, *Npr3*^*high*^) also showed high expression of *PPARg*^*+*^ and *Fabp4*^*+*^, suggesting that they were likely captured during their differentiation into mature adipocytes (Fig. 1F,H). Supporting this hypothesis, this cluster also displayed the lowest expression of *Pdgfra* and *Dlk1* (Fig. 1H). However, because their overall transcriptional signature closely aligns with wFAPLs, we named this cluster differentiating wFAPLs (Fig. 1F,H).

**Figure EV2:**

WAT and BAT APC display unique transcriptional signatures. (**A**) Heatmap of the top 10% differentially expressed genes for each white APC cluster. (**B**) Heatmap of the top 10% differentially expressed genes for each brown APC cluster.

Similarly, each BAT cluster had a unique set of differentially expressed genes (Fig. EV2B; Dataset EV2). Of them, five BAT clusters (bAPC 1, *Sema3a*^*high*^, *Nav3*^*high*^, 26.8%; bAPC 2, *Lrrc15*^*+*^, 21.5%; bAPC 3, *Cdca3*^*+*^, *Kif20a*^*+*^, 9.85%; bAPC 4, *Creb5*^*+*^, *Ptprz1*^*+*^, 7.42%; and bAPC 5, *Igf1*^*high*^, *6%)* showed low expression of *Pparg* and its target genes (e.g., *Fabp4*) and high *Dlk1* levels, suggesting an early stage of brown adipose tissue progenitors (Fig. 1G,I). As observed for WAT progenitors, one BAT population showed higher expression of *Pparg* and *Fabp4* (*Pparg*^*high*^, *Fabp4*^*high*^) and represented a cluster of brown committed adipocyte precursors (bCAPs) (Fig. 1G,I). Interestingly, in BAT, a cluster representing ~15% of the total cell population was characterized by low *Pparg* and *Fabp4* levels and high expression of ECM/stress/immunomodulatory genes (*Serpinb1a*^*+*^, *Cryab*^*+*^). Given their similarity to wFAPLs, we named this cluster brown Fibro-Adipogenic-Progenitor-Like cells (bFAPLs) (Fig. 1G,I).

### WAT progenitors display two distinct developmental trajectories in vitro

**Figure 2:**
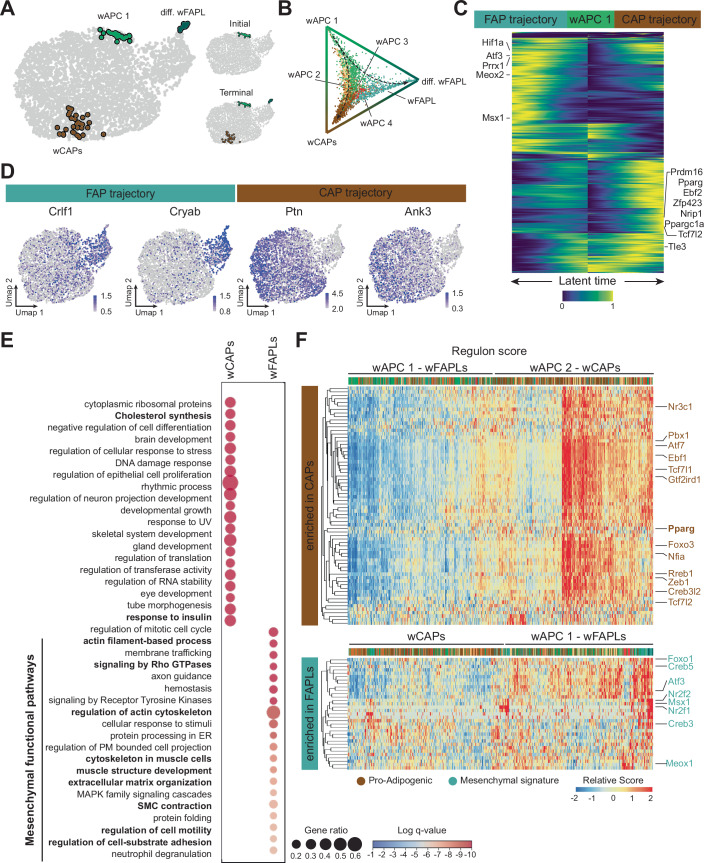
Developmental trajectories of WAT progenitors. (**A**) CellRank analysis reveals three macrostates: wAPC1, wCAPs, and differentiating wFAPLs. While all three states identified as terminal states (bottom right), only wAPC 1 is also identified as initial state (top right). (**B**) Fate probabilities for each cell towards the three terminal states, highlighting wCAPs and wFAPLs as true distinct terminal lineages. (**C**) Heatmap of the driver genes in wCAP and wFAPL trajectories, computed via generalized additive modeling (GAM). Genes are ordered by their temporal activation patterns along inferred latent time. (**D**) Feature plots of representative drivers overlapping with wCAPs and wFAPLs DEGs, respectively, underscore the divergence between wFAPLs and wCAPs fate. (**E**) Biological pathway analysis of differentially expressed genes between wCAPs vs wFAPLs reveals enrichment in metabolic and insulin-responsive pathways in wCAPs, while mesenchymal-related pathways are enriched in wFAPLs. (**F**) Hierarchical heatmaps of regulon scores centering transcription factors enriched in wCAPs (top, defined by wCAP differential scores) and wFAPLs trajectories (bottom, defined by wFAPL differential scores), showing alignment of subclusters to their respective fates. Source data are available online for this figure.

To determine whether white progenitor clusters constitute discrete cell types or different transition states of the same progenitors, we performed pseudotime analysis to reconstruct the differentiation trajectories of white preadipocytes using RNA velocity ScVelo (Bergen et al, 2020) and define macrostates using CellRank (Lange et al, 2022). ScVelo identified two major differentiation paths: one leading to wCAPs through wAPC2, and the other to differentiating wFAPLs via wFAPLs (Fig. EV3A). In addition, CellRank identified three macrostates, consisting of wAPC1, wCAPs, and differentiating wFAPLs (Figs. 2A and  EV3B). Notably, all three macrostates exhibited metastability scores (i.e., the likelihood to remain in a given state short-term) consistent with terminal state classification (Fig. EV3C). However, the reduced tendency of wAPC 1 to remain in that state in the long-term, indicated by its low stationary distribution, also identified this cluster as the only initial macrostate (Figs. 2A and EV3C). This suggests that the wAPC 1 cluster may serve as a reservoir of proliferating adipocyte progenitors capable of either maintaining an undifferentiated state (i.e., remaining as wACP 1) or progressing along the pseudotime trajectory towards wCAPs via the intermediate state wAPC 2 or towards differentiating wFAPLs via their earlier state wFAPLs (Figs. 2B and EV3D).

**Figure EV3:**

Pseudotime trajectory inferences and macrostate assignments in WAT. (**A**) Latent time UMAP visualization inferred by ScVelo and model of WAT APC differentiation trajectories highlight the transition from early precursors (wAPC 1) to wCAPs and wFAPLs. (**B**) Composition of WAT APC macrostates (*n* = 30 cells) by CellRank2. (**C**) Coarse-grained transition matrix using CellRank2 GPCCA for WAT macrostates indicating the stability (diagonal elements) and transition probabilities (stationary distribution) amongst macrostates. (**D**) Proposed developmental model of WAT APCs shows a shared early progenitor wAPC 1 that can differentiate into wFAPLs or wCAPs via intermediate transition states (wAPC 2). (**E**) Biological pathway analysis of the transcription factor drivers of wCAPs and wFAPLs shows enrichment in adipogenesis for wCAPs and mesenchymal-related pathways for wFAPLs.

Having identified the initial and terminal fates, we next sought to visualize gene expression dynamics along these lineages to uncover key regulatory factors. To accomplish this, we computed putative driver genes for each of the two fates (Dataset EV3), plotting them side by side based on their temporal ordering (Fig. 2C). Perhaps not surprisingly, many wCAPs and wFAPLs drivers overlapped with their respective differentially expressed genes, such as *Cryab* and *Crlf1* for wFAPLs, or *Ptn* and *Ank3* for wCAPs (Fig. 2D**)**. Interestingly, 227 transcriptional regulators (8.4% of total drivers), including known regulators of adipogenesis such as *Pparg*, *Prdm16*, *Tcf7l2*, *Tle3*, *Zfp423*, *Ebf2*, *Nrip1*, and *Ppargc1a*, were observed amongst the drivers of wCAPs (Figs. 2C and EV3E; Dataset EV3). Conversely, only 112 transcriptional regulators (5.1% of total drivers), mostly linked to mesenchymal cell proliferation and differentiation, were found as drivers of differentiating wFAPLs (Figs. 2C and EV3E; Dataset EV3).

To further characterize cluster-defining genes, we performed biological pathway analysis of genes differentially expressed between wCAPs and wFAPLs (Fig. 2E; Dataset EV3). Classical adipocyte markers, including *Pparg* and *Fabp4*, insulin growth factors *Igf1* and *Igf2*, and lipid metabolism-related genes such as *Lpl* and *Cyp7b1* were significantly higher in wCAPs, whereas the stress response protein *Cryab*, the ECM-related genes *Col4a1*, *Col4a2*, and *Serpinb1a*, the anti-adipogenic mesenchyme homeobox *Meox2*(Liu et al, 2015), and other inflammatory-related genes, including *Crlf1* and *Cxcl14*, were higher in wFAPLs and/or differentiating wFAPLs, strongly suggesting a mesenchymal transcriptional signature (Dataset EV3). More globally, wCAPs were enriched for metabolic pathways such as cholesterol synthesis and response to insulin, both hallmarks of mature white adipocytes (Fig. 2E). Conversely, wFAPLs displayed pathways linked to extracellular matrix organization, signaling to Rho GTPases, regulation of the actin cytoskeleton, muscle structure development, smooth muscle cell contraction (Fig. 2E), processes that have been previously shown to impact expansion of white fat in response to HFD and inflammation(Sun et al, 2023) and that confirm the mesenchymal identity of this subpopulation.

Finally, to gain further insights into the transcriptional networks driving wCAPs and wFAPLs differentiation, we calculated the activity score of transcription factors based on co-expression modules of their putative target genes, also called regulons, using SCENIC (Aibar et al, 2017). Notably, wCAPs were characterized by high regulon activity of Pparγ as well as several other pro-adipogenic transcription factors (Nr3c1, Ebf1, Tcf7l1, Tcf7l2, Zeb1, Creb3l2) and known modulators of Pparγ transcriptional activity (Pbx1, Atf7, Gtf2ird1, Foxo3, Nfia, Rreb1) (Fig. 2F). Conversely, wFAPLs exhibited a core set of inflammation-related and mesenchymal fate determination regulons, including Creb3 and Creb5, Foxo1, Atf3, Nr2f1 and Nr2f2, Meox1, and Msx1 (Fig. 2F), strongly supporting their mesenchymal identity. Furthermore, consistent with the inferred developmental trajectories calculated via RNA velocity (Fig. EV3A), hierarchical clustering of regulon activity revealed a clear separation in lineage commitment: wAPC 2 preferentially grouped with wCAPs, consistent with its role as a transitional intermediate in the adipogenic trajectory, while wAPC 1 clustered more closely with wFAPLs, reflecting a more direct progression toward the fibroadipogenic lineage (Fig. 2F). These results support a developmental model for APCs in WAT in which a dormant fibrogenic progenitor population (wAPC 1) can diverge into two distinct developmental trajectories: one leading to committed adipogenic precursors (wCAPs) via intermediate states (e.g., wAPC 1 to wAPC2 to wCAPs) and the other one leading directly to fibroadipogenic-like cells (wAPC1 to wFAPLs and differentiating wFAPLs) (Fig. EV3D).

Altogether, our data indicate that our in vitro model can capture cell heterogeneity of adipocyte progenitors concordant with known preadipocyte subtypes previously described and, therefore, this model can be leveraged to investigate cell state transition.

### Primary BAT progenitors mirror the developmental trajectories of their white preadipocyte counterparts

**Figure 3:**
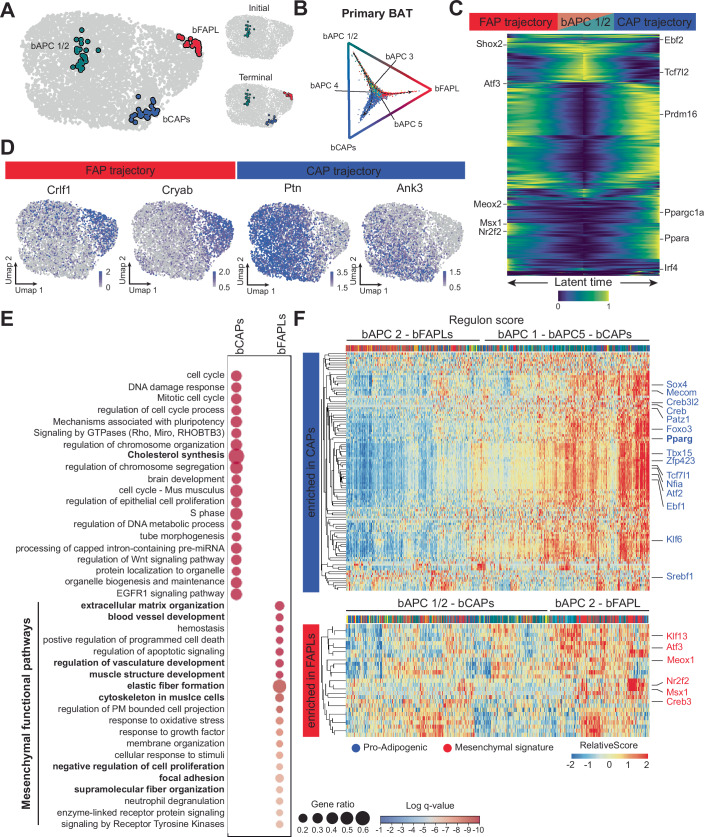
Developmental trajectories of BAT progenitors. (**A**) CellRank analysis reveals three macrostates: bAPC 1/2, bCAPs, and bFAPLs. While all three states identified as terminal states (bottom right), only bAPC 1/2 is also identified as an initial state (top right). (**B**) Fate probabilities for each cell towards the three terminal states, highlighting bCAPs and bFAPLs as true distinct terminal lineages. (**C**) Heatmap of the driver genes in bCAP and bFAPL trajectories, computed via GAM. Genes are ordered by their temporal activation patterns along inferred latent time. (**D**) Feature plots of representative drivers overlapping with bCAPs and bFAPLs DEGs, respectively, underscore the divergence between bFAPLs and bCAPs fate. (**E**) Similar to WAT APCs, biological pathway analysis of differentially expressed genes between bCAPs vs bFAPLs shows metabolic pathways in bCAPs, and a mesenchymal signature in bFAPLs. (**F**) Hierarchical heatmaps of regulon scores centering transcription factors enriched in bCAPs (top, defined by bCAPs differential scores) and bFAPLs trajectories (bottom, defined by bFAPLs differential scores), showing alignment of subclusters to their respective fates. Source data are available online for this figure.

**Figure EV4:**
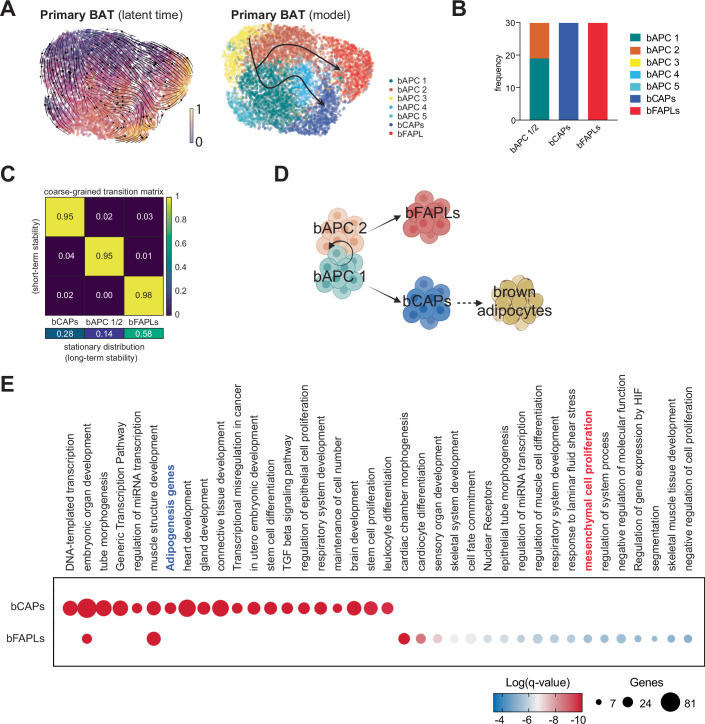
Pseudotime trajectory inferences and macrostate assignments in BAT. (**A**) Latent time UMAP visualization inferred by ScVelo and model of BAT APC differentiation trajectories highlight the transition from early precursors (bAPCs 1/2) to bCAPs and bFAPLs. (**B**) Composition of BAT APC macrostates (*n* = 30 cells) by CellRank2. (**C**) Coarse-grained transition matrix using CellRank2 GPCCA for WAT macrostates indicating the stability (diagonal elements) and transition probabilities (stationary distribution) amongst BAT macrostates. (**D**) Proposed developmental model of BAT APCs shows a mixed population of bAPC 1 and bAPC 2 that can differentiate into bCAPs and bFAPLs, respectively. (**E**) Biological pathway analysis of the transcription factor drivers of bCAPs and bFAPLs shows enrichment in adipogenesis for bCAPs and mesenchymal-related pathways for bFAPLs.

After validating the heterogeneity of WAT preadipocytes, we applied the same computational pipeline to determine the developmental trajectories of the less characterized BAT progenitors. Similar to WAT, we observed two distinct differentiation paths for bCAPs and bFAPLs (Fig. EV4A). CellRank identified three macrostates representing bCAPS, bFAPLs, and a mixed population of bAPC 1 and bAPC 2 (Figs. 3A,B and EV4B), indicating that these two clusters may be sufficiently similar to unify into a single macrostate. As found for wAPC 1, the bAPC 1/2 macrostate was assigned both initial and terminal state (Fig. 3A,B) based on its metastability and stationary distribution (Fig. EV4C), hinting at bAPC 1 and bAPC 2 as recruitable sources supporting bCAPs and bFAPLs maintenance, while bAPC 3–5 depict intermediate transition states (Figs. 3B and EV4D).

Differentially expressed genes and driver gene modules for bCAPs and bFAPLs reflected the differences found in WAT progenitors (Fig. 3C,D; Dataset EV4). The drivers of bCAPs were enriched in pro-adipogenic and thermogenic regulators, such as *Ppara*, *Prdm16*, *Ebf2*, *Irf4*, and *Ppargc1a* (Figs. 3C and EV4E; Dataset EV4), whereas drivers of bFAPLs showed mesenchymal and skeletal muscle regulators, including *Msx1*, *Meox1*, *Shox2*, *Nr2f2*, and *Atf3* (Figs. 3C and EV4E; Dataset EV4). Several wFAPLs drivers were also found to be significantly higher in bFAPLs, including *Cryab*, *Meox2*, *Col4a1*, *Col4a2*, *Crlf1*, *Serpinb1a*, and *Nr2f2*, indicating that BAT also possesses a distinct mesenchymal-like progenitor population with fibroadipogenic characteristics. Consistent with this observation, biological pathway analysis of differentially expressed genes between bCAPs and bFAPLs showed a marked mesenchymal signature of bFAPLs, with ECM remodeling and vasculature development processes (Fig. 3E; Dataset EV4). On the other hand, in contrast to wCAPs, bCAPs showed only moderate enrichment in metabolic pathways and a stronger signature related to development, cell cycle, and organelle biogenesis and organization (Fig. 3E; Dataset EV4).

Finally, regulon analysis showed a remarkable similarity with WAT progenitors. Pro-adipogenic transcription factors, including Pparγ, Creb, Tcf7l1, Creb3l2, Atf2, Klf6, Zfp423, Ebf1, Patz1, and Tbx15, and known regulators of Pparγ expression (Sox4, Mecom, Foxo3, Nfia) scored highly in bAPC 1 and bCAPs (Fig. 3F), whereas inflammation- and mesenchymal-related transcription factors, including Msx1, Meox2, Creb3, Atf3, Nr2f2, and Klf13 were enriched in bAPC 2 and bFAPL transcriptional signatures (Fig. 3F). Collectively, our data support a developmental model for APCs in BAT in which bAPC 1 and bAPC 2 represent early stages of proliferating brown adipocyte progenitors that can differentiate into committed brown adipogenic precursors (bCAPs) and fibroadipogenic-like cells (bFAPLs), respectively (Fig. EV4D). Most notably, our results indicate that both WAT and BAT show remarkably similar developmental trajectories and highlight the presence of a previously unappreciated fibroadipogenic population in BAT.

### WAT and BAT FAPLs are transcriptionally related to each other

**Figure 4:**
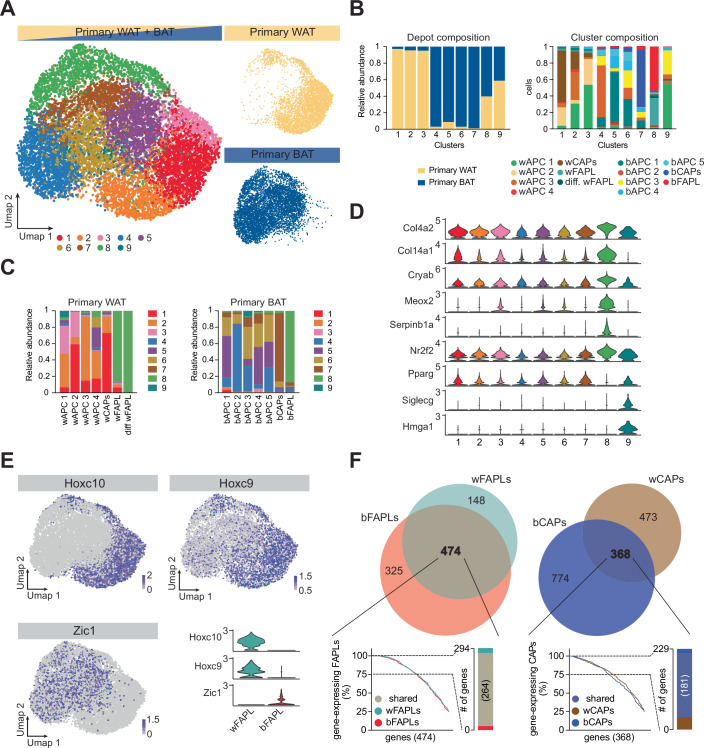
WAT and BAT precursors share a common population of fibroadipogenic cells. (**A**) UMAP projection of combined WAT and BAT APCs after cell cycle regression reveals nine clusters. Small UMAPs indicate how WAT and BAT cells are distributed. (**B**) Quantification of white and brown APCs across each combined cluster. Clusters 8 and 9 are characterized by equal representation of WAT and BAT cells. (**C**) Quantification of how aggregated clusters compose white and brown APC clusters shows that cluster 8 is made of FAPL progenitors from both WAT and BAT. (**D**) Cluster 8 markers reflect depot-specific FAPL markers (*n* = 10,289 cells in total for WAT and BAT aggregate). (**E**) Expression of depot-specific genes such as *Hoxc10* and *Hoxc9* for WAT, and *Zic1* for BAT demonstrate the absence of cross-contamination of white progenitors in BAT progenitors and vice-versa (*n* = 10,289 cells). (**F**) Venn diagrams representing the overlap of genes differentially expressed between wFAPLs and bFAPLs, and between wCAPs and bCAPs. Genes shared between white and brown populations were ranked by the percentage of cells expressing each gene within their respective clusters. Genes expressed in 75% or more of wFAPLs, bFAPLs, wCAPs, and bCAPs were then categorized as white-only (genes meeting the threshold only in wFAPLs or wCAPs), brown-only (genes meeting the threshold only in bFAPLs or bCAPs), or shared (genes meeting the threshold in both white and brown FAPLs or CAPs). Source data are available online for this figure.

**Figure EV5:**
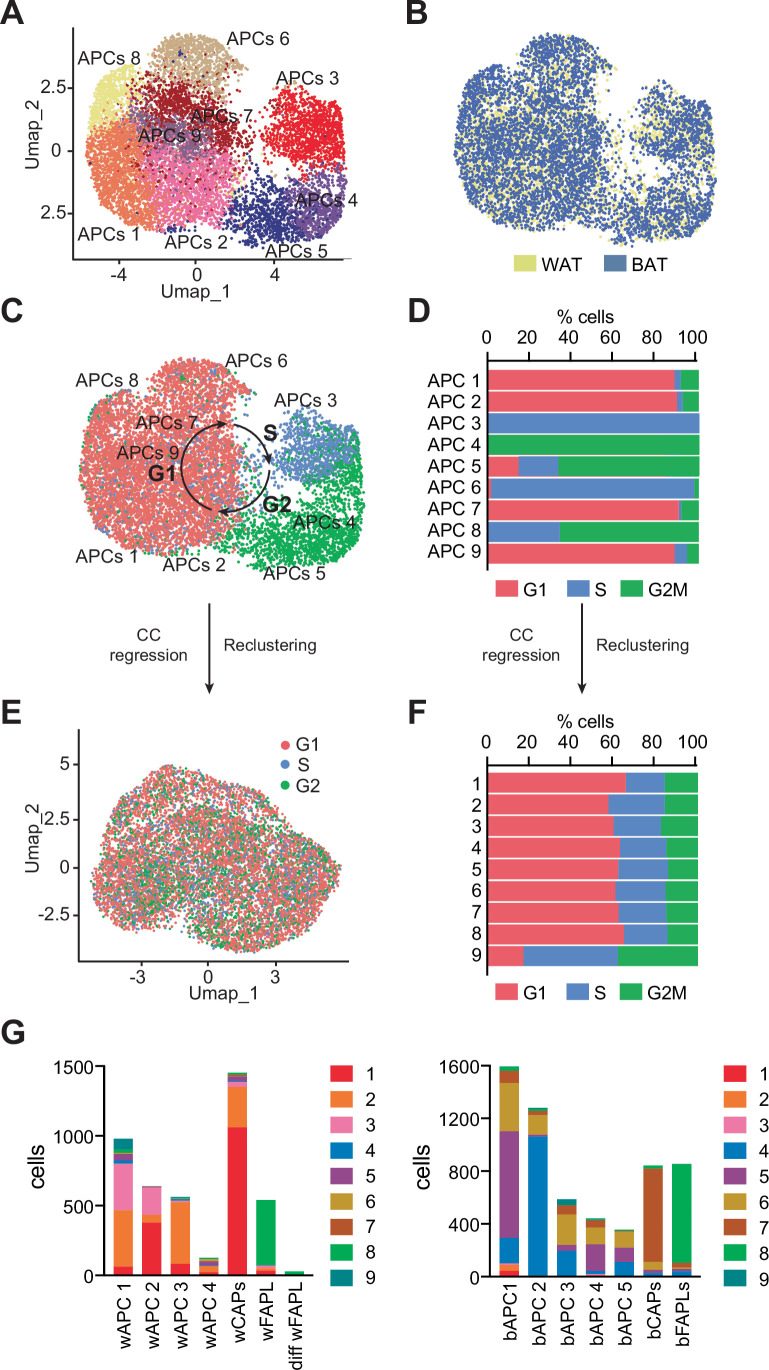
Integrated analysis of WAT and BAT precursor populations. (**A**) Clustering of integrated WAT and BAT APCs before cell cycle regression. (**B**) Distribution of WAT and BAT APCs in the aggregate UMAP. (**C**, **D**) Cell cycle phase (G1, S, G2/M) of WAT and BAT APCs and distribution across clusters. (**E**) UMAP of WAT and BAT APC aggregate after cell cycle regression. (**F**) Quantification of cell cycle frequency across clusters. (**G**) Cell count of white and brown APCs across each combined cluster.

To explore regulatory and developmental parallels between white and brown adipocyte precursors, we combined the two datasets into a unified aggregate. As before, we applied cell cycle regression (Fig. EV5A–F) and identified nine clusters (Fig. 4A). Three of these clusters (1–3) were predominantly composed of white progenitors (Figs. 4B and  EV5G), four clusters were heavily enriched in brown progenitors (4–7), and two clusters (8 and 9) showed even brown/white cells distribution (40% WAT and 60% BAT in cluster 8, and 59% WAT and 41% BAT in cluster 9, respectively) (Figs. 4B and  EV5G). Notably, cluster 1 was composed for the most part (63%) of wCAPs and their earlier progenitors wAPC 2 (22%), while bCAPs (70%) and bAPC 1 (9%) constituted the majority of cluster 7 (Figs. 4B,C and EV5G). Of the 2 mixed clusters, cluster 9 only included a small number of cells (145 cells total) coming from wAPC 1 (54%), wAPC 3 (4%), and bAPC 1–4 (2%, 6%, 29%, and 4%, respectively) (Figs. 4B,C and EV5G). Interestingly instead, cluster 8 was almost entirely made of white and brown FAPLs (34% and 54%, respectively) (Figs. 4B,C and EV5G), further supporting that these fibroadipogenic-like cells from white and brown adipose depots are remarkably similar. Because cluster 8 was mainly made of FAPLs, its distinct markers included ECM- and stress-related genes such as *Cryab, Meox2, Col4a1, Col4a2, Nr2f2*, and *Serpinb1a*, as seen for white and brown FAPLs independently (Fig. 4D), as well as low levels of classical adipogenic markers such as *Pparg* (Fig. 4D). Importantly, *Hoxc9* and *Hoxc10*, transcription factor members of the *Hox* family of homeobox genes highly expressed in WAT, but not BAT (Brune et al, 2016), were detectable in wFAPLs but not bFAPLs (Fig. 4E). Conversely, the highly specific marker of interscapular BAT, the transcription factor *Zic1* (Walden et al, 2012), was expressed in bFAPLs but not wFAPLs (Fig. 4E). The detection of depot-specific markers confirmed the absence of cross-contamination between adipocyte populations, addressing concerns associated with the isolation of primary cells, particularly brown preadipocytes, due to the surrounding white adipose tissue. These results ruled out the possibility that bFAPLs originated from co-isolated white adipocyte progenitors and reinforced the conclusion that bFAPLs represent a distinct, bona fide progenitor population within brown adipose tissue. These observations support the notion that, despite arising from depot-specific progenitors, white and brown FAPLs undergo converging developmental programs that culminate in a shared cell identity defined by common functional and molecular traits. In fact, despite the majority (474 genes) of FAPL markers being shared between white and brown FAPLs (Fig. 4F; Dataset EV5), analyses of genes differentially expressed in white and brown FAPLs also revealed 148 white- and 325 brown-specific markers (Fig. 4F; Dataset EV5). Conversely, less markers were shared between white and brown CAPs (473 wCAP-specific, 774 bCAP-specific, and 368 shared), which can also explain why FAPLs clustered together while CAPs segregated into distinct clusters when white and brown progenitors are aggregated.

To define a robust and representative set of marker genes for FAPLs and CAPs, we focused on those shared between white and brown depots. To ensure that the selected genes reliably characterize each population, we calculated the percentage of cells expressing each gene within each cluster (Fig. 4F). Using a stringent threshold, we selected only genes expressed in 75% or more of the cells within each respective population. This analysis identified 294 genes for FAPLs and 229 for CAPs. Of these, 264 genes (89.7%) were expressed in ≥75% of cells in both wFAPLs and bFAPLs, and 181 genes (79%) met the same criterion in both wCAPs and bCAPs (Dataset EV6). These results indicate the presence of broadly expressed, depot-independent transcriptional signatures that define the FAPL and CAP preadipocyte subtypes.

Together, these findings indicate that early white and brown progenitors diverge into two distinct lineages: a depot-specific committed adipogenic precursor state (wCAPs/bCAPs), and a shared fibroadipogenic-like state (wFAPLs/bFAPLs) exhibiting a highly conserved transcriptional signature across depots.

### Cultured APCs recapitulate the heterogeneity of APCs in vivo

**Figure EV6:**
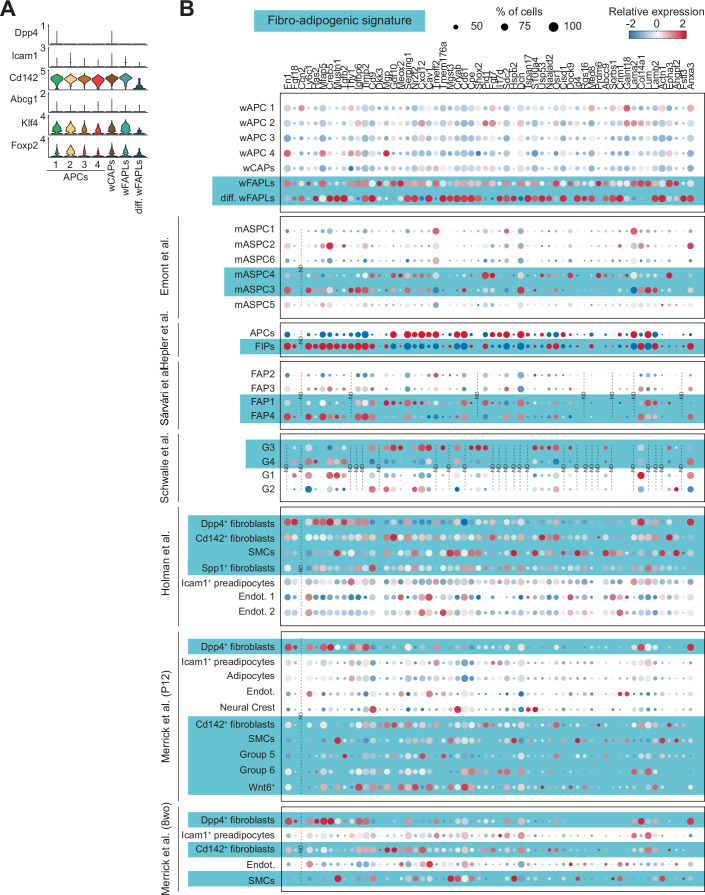
Identification of a shared FAP signature across multiple datasets. (**A**) Expression in culture WAT APCs of known genes previously reported to define mASPC4, FIP, *Dpp4*^*+*^, *Icam1*^*+*^, *Cd142*^*+*^, Aregs and FAP populations in the WAT APC datasets (*n* = 4331 cells). (**B**) Expression of other differentially expressed genes enriched in wFAPLs and differentiating wFAPLs compared to the expression found in previously reported fibroadipogenic populations.

To assess whether our in vitro observations align with previously characterized APC populations, we compared our datasets to previously published scRNA-seq/snRNA-seq studies of similar precursor cells (Dara ref: (Burl et al, 2018; Emont et al, 2022; Hepler et al, 2018; Holman et al, 2024; Karlina et al, 2021; Merrick et al, 2019; Sarvari et al, 2021; Schwalie et al, 2018)). Somehow surprisingly, some reported markers of adipocyte progenitors, including *Dpp4* and *Icam1* (Merrick et al, 2019), *CD142/F3* and *Abcg1* (Schwalie et al, 2018), or *Klf4* and *Foxp2* (Sarvari et al, 2021), were either expressed at very low levels or not significantly different amongst clusters (Fig. EV6A). Nevertheless, differentially expressed genes in wFAPLs/differentiating wFAPLs showed substantial qualitative overlap with numerous differentially expressed genes observed in previous studies for mASPC3/mASPC4 (Emont et al, 2022), FIPs (Hepler et al, 2018), FAP1/4 (Sarvari et al, 2021), G3 (Aregs)/G4 (Schwalie et al, 2018), and Dpp4^+^, *Cd142*^+^, or *Spp1*^+^ fibroblasts (Holman et al, 2024; Merrick et al, 2019) (Fig. EV6B). Among them, *Fn1*, *Ly6c1*, *Mfap5*, *Creb5*, *Timp2*, *Cd9*, and *Igfbp6* appeared to be consistently elevated in the above-mentioned fibroadipogenic populations (Fig. EV6B).

**Figure 5:**
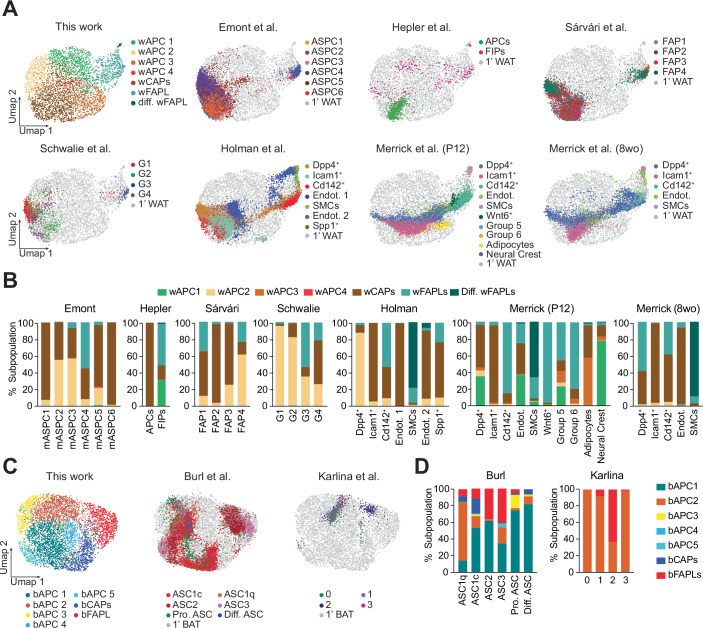
Cultured APCs recapitulate the heterogeneity of APCs found in vivo. (**A**) Reference mapping of previously published datasets against primary WAT progenitors links known mesenchymal populations to wFAPLs while highly adipogenic subpopulations map to wCAPs. (**B**) Quantification of reference mapping distribution for WAT datasets. (**C**) Reference mapping of previously published datasets against primary BAT progenitors reveals a fibrogenic population of brown adipose tissue progenitors in vivo. (**D**) Quantification of reference mapping for BAT datasets. Source data are available online for this figure.

Next, to globally assess the similarity of cultured white and brown progenitors with their counterparts identified in vivo, we performed reference mapping using precalculated PCA as integration anchors from our dataset against published datasets (Data ref: (Burl et al, 2018; Emont et al, 2022; Hepler et al, 2018; Holman et al, 2024; Karlina et al, 2021; Merrick et al, 2019; Sarvari et al, 2021; Schwalie et al, 2018)). Notably, our wFAPL population shared high qualitative overlap with previously described FAP or FAP-like cells, namely mASPC3 (*Mgp*^+^) and mASPC4 (*Epha3*^*+*^) (Emont et al, 2022), FIPs (*Ly6c1*^*+*^) (Hepler et al, 2018), FAP4 (*Klf4*^+^) and FAP1 (*Foxp2*^+^) (Sarvari et al, 2021), G3/Aregs (Cd142^+^) and G4 (Ly6c1^+^) (Schwalie et al, 2018), and Dpp4^+^ and Cd142^+^ fibroblasts (Holman et al, 2024; Merrick et al, 2019) (Fig. 5A,B), indicating that cultured wFAPLs recapitulate the mesenchymal, fibroadipogenic identity observed in vivo. Similarly, highly adipogenic mASPC1, mASPC5, and mASPC6 (Emont et al, 2022), APCs (Hepler et al, 2018), FAP2 and 3 (Sarvari et al, 2021), G1 and G2 (Schwalie et al, 2018), and *Icam1*^+^ preadipocytes (Holman et al, 2024; Merrick et al, 2019) mapped to wCAPs or their precursors wAPC 2 (Fig. 5A,B), confirming the pro-adipogenic nature of cultured wAPC 2 and wCAPs. Interestingly, our early mesenchymal-like cluster, wAPC 1, was only matched by FAP counterparts in datasets from postnatal (P12) mice (Merrick et al, 2019) or young adults (6-week-old) (Hepler et al, 2018) (Fig. 5B). In contrast, in datasets from mice aged 8 weeks or older, wAPC 1 was entirely absent and replaced by wFAPLs (Fig. 5B), suggesting that early mesenchymal stem cells progressively transition into more committed progenitor states with age.

Fewer datasets have been generated for BAT progenitors, which have remained to date less thoroughly characterized. Despite this, we found that bCAPs strongly aligned with the previously reported cold-responsive ASC1 progenitors (Burl et al, 2018), while bFAPLs overlap in part with the vasculature-associated ASC2/ASC3 (Burl et al, 2018) (Fig. 5C,D). Similarly, the uncharacterized cluster 2 from another, independent study (Karlina et al, 2021) showed significant enrichment (~60%) of bFAPL-like cells, while all the other clusters (0, 1, and 3) mapped onto bAPC 2, the earlier progenitor of bFAPLs. These results demonstrate that cultured WAT and BAT APCs align with previous in vivo phenotypes and that the heterogeneous APC populations isolated from neonatal pups provide a valuable model for investigating distinct adipocyte lineages.

## Discussion

Understanding the cellular heterogeneity of adipose tissue progenitors is central to dissecting the mechanisms regulating adipose tissue development, remodeling, and function. While single-cell RNA-sequencing has recently advanced our knowledge of progenitor populations in vivo (Duerre and Galmozzi, 2022; Loft et al, 2025; Maniyadath et al, 2023), in vitro systems that faithfully recapitulate this heterogeneity have remained limited. In this study, we generated single-cell transcriptomic profiles from cultured progenitors derived from both white and brown adipose tissue depots. Our data reveal that key features of adipose progenitor diversity are preserved in vitro, providing a powerful tool to bridge in vivo findings with mechanistically tractable systems.

A central finding of our work is the resolution of two main progenitor trajectories that are maintained during early in vitro culture: committed adipogenic precursors (CAPs) and fibroadipogenic progenitor-like cells (FAPLs). These populations are transcriptionally distinct and resemble the bifurcating lineages that have been consistently described in vivo (Duerre and Galmozzi, 2022; Maniyadath et al, 2023). Importantly, we demonstrate that both white and brown adipose tissue give rise to these two progenitor trajectories, indicating that the underlying lineage programs are conserved across depots. The presence of both adipogenic and fibroadipogenic lineages under standard culture conditions suggests that in vitro models retain a level of progenitor plasticity and heterogeneity relevant for studying adipose tissue physiology.

Most notably, our study reveals a previously undescribed progenitor population in BAT with strong transcriptional similarity to fibroadipogenic, mesenchymal progenitors found in WAT. FAPs have been well characterized in WAT (Hepler et al, 2018; Merrick et al, 2019; Sarvari et al, 2021; Schwalie et al, 2018) and skeletal muscle (Joe et al, 2010; Uezumi et al, 2010; Wosczyna et al, 2019; Yang et al, 2022), where they contribute to extracellular matrix production and fibrosis, but had not been described in BAT prior to this study. Using our in vitro model and reference mapping to in vivo datasets (Burl et al, 2018; Karlina et al, 2021), we showed that brown adipose FAPs, despite originating from distinct lineage-specific progenitors, are remarkably similar to white adipose FAPs and are present within brown adipose tissue in vivo, highlighting the utility of our system in uncovering adipocyte progenitor types that may be rare, transient, or previously overlooked in complex in vivo environments.

Our dataset also allows alignment of in vitro-derived populations with known in vivo progenitor classes, including CAPs, Dpp4^+^ interstitial progenitors, FIPs, FAPs, and Aregs. Although APCs dynamically change with aging and pathophysiology (Merrick et al, 2019; Rondini et al, 2021; Sarvari et al, 2021; Sun et al, 2020; Yang et al, 2022; Zhang et al, 2022), and a direct equivalence between in vitro and in vivo populations must be interpreted with caution, the consistency in gene expression patterns that we observed between cultured APCs and their in vivo counterparts supports the relevance of this in vitro system for modeling adipose tissue development and dynamics.

In addition to the core CAP and FAPL lineages, our analysis identified progenitor populations such as wAPC 1 that align with early-stage, multipotent progenitors enriched in neonatal tissue. These populations share transcriptional features with progenitor subtypes observed in younger animals, such as FIPs (Hepler et al, 2018), *Dpp4⁺* fibroblasts and endothelial cells (Merrick et al, 2019), and appear to diminish with age, suggesting a developmental progression toward more committed states (i.e., FAPLs). Moreover, our trajectory and metastability analyses revealed that certain populations, including wAPC3 and wAPC 4, likely represent transitional phases between progenitor states. These intermediate phases may be more readily resolved under in vitro conditions, where reduced environmental and cellular complexity allows for the detection of subtle lineage transitions that are difficult to capture in vivo. As such, in vitro systems may offer a valuable window into dynamic processes that are otherwise transient or obscured in native tissues. In this context, our results validate a practical and scalable model for dissecting the regulatory mechanisms that govern adipose progenitor fate. Unlike immortalized cell lines, primary cells capture the heterogeneity of APCs in vivo and can be confidently used to study how environmental cues or signaling pathways influence adipogenic versus fibroadipogenic fate. In addition, supporting current efforts to define a broadly accepted nomenclature for adipocyte precursors (Loft et al, 2025; Maniyadath et al, 2023), this resource might facilitate the identification of shared markers, which may be used for isolation and targeted functional studies, facilitating translational insights into depot-specific remodeling and metabolic disease.

In summary, our study establishes an in vitro single-cell atlas of white and brown adipose progenitors that recapitulates key features of in vivo biology. By enabling the identification of brown FAPs and providing a framework to study progenitor heterogeneity in controlled conditions, this work offers a valuable resource for the adipose biology community and a platform for mechanistic exploration of lineage dynamics.

## Methods

**Table:** Reagents and tools table

Reagent/resource	Reference or source	Identifier or catalog number
**Experimental models**		
C57BL6/J (*M. musculus*)	Jackson Labs	JAX# 000664
**Chemicals, enzymes, and other reagents**		
DMEM	Gibco	11965-092
Sodium chloride	Sigma-Aldrich	S7653
Potassium chloride	Sigma-Aldrich	P9333
Calcium chloride	Sigma-Aldrich	C4901
D-(+)-Glucose	Sigma-Aldrich	G70121
HEPES	Gibco	15630-080
Bovine serum albumin	Sigma-Aldrich	A7030
Antibiotic-antimycotic	Gibco	15240-062
DPBS	Gibco	14190-144
Fetal bovine serum	Gemini Bio	100-106
Sodium pyruvate	Gibco	11360-070
GlutaMAX supplement	Gibco	35050-061
Collagenase, Type 1	Worthington Biochemical	LS004196
Gelatin from porcine skin	Sigma-Aldrich	G1890
Trypsin-EDTA (0.05%)	Gibco	25300-054
Trypan Blue	Sigma-Aldrich	T8154
**Software**		
GraphPad Prism 10.0.1		RRID:SCR_002798 https://www.graphpad.com/
R programming language v4.4.1	R core	RRID:SCR_001905 https://www.r-project.org/
R package Seurat 5.1.0	Hao et al, 2024	RRID:SCR_016341 https://satijalab.org/seurat/
R package dplyr	Wickham et al, 2025	RRID:SCR_016708 https://dplyr.tidyverse.org/
R package ggplot2 3.5.1	Wickham, 2016	RRID:SCR_014601 https://ggplot2.tidyverse.org/index.html
R package cowplot v1.1.3	Wilke C, 2024	RRID:SCR_018081 https://wilkelab.org/cowplot/index.html
R package scCustomize v2.1.2	Marsh, 2021	RRID:SCR_024675 https://samuel-marsh.github.io/scCustomize/index.html
R package pheatmap	Kolde R, 2025	RRID:SCR_016418 https://github.com/raivokolde/pheatmap
R package sctransform v0.4.1	Hafemeister and Satjia, 2019; Choudhary and Satjia, 2022	RRID:SCR_022146 https://github.com/satijalab/sctransform
pySCENIC	Aibar et al, 2017	RRID:SCR_017247 https://github.com/aertslab/SCENIC
pyGPCCA	Reuter et al, 2019	https://github.com/msmdev/pyGPCCA
Python 3.9.12	Python Software Foundation	RRID:SCR_008394 https://www.python.org/downloads/
Python module anndata v0.8.0	Virshup et al, 2024	RRID:SCR_018209 https://github.com/scverse/anndata
Python module CellRank2	Lange et al, 2022	RRID:SCR_022827 https://cellrank.readthedocs.io/en/latest/index.html
Python module igraph 0.10.3	Csardi G and Nepusz T, 2006	RRID:SCR_019225 https://python.igraph.org/en/stable/
Python module matplotlib	Hunter, 2007	RID:SCR_008624 https://matplotlib.org/stable/
Python module numpy	Harris, 2020	RRID:SCR_008633 https://numpy.org/install/
Python module pandas v1.5.2	The pandas development team, 2020; McKinney, 2010	RRID:SCR_018214 https://pandas.pydata.org/
Python module scanpy v1.9.1	Wolf et al, 2018	SCR_018139 https://github.com/theislab/scanpy
Python module scvelo v0.2.5	Bergen et al, 2020	RRID:SCR_018168 https://github.com/theislab/scvelo
Python module seaborn 0.12.2	Waskom ML, 2021	RRID:SCR_01813 https://seaborn.pydata.org/
Python module loompy v3.0.7	Linnarsson Lab	RRID:SCR_016666 https://loompy.org
Cell Ranger 7.1.0	10x Genomics Cloud Analysis	RRID:SCR_017344 https://www.10xgenomics.com/products/cloud-analysis
**Other**		
Brown adipose tissue scRNAseq	Burl et al, 2018https://github.com/RBBurl1227/eLife-2022-ColdInducedBrownAdipocyteNeogenesis	GSE207707
Brown adipose tissue SVF scRNAseq	Karlina et al, 2021	GSE161447
Visceral white adipose tissue Pdgfrβ+ cells scRNAseq	Hepler, 2018	GSE111588
Subcutaneous white adipose tissue SVF scRNAseq	Schwalie et al, 2018	E-MTAB-5802
Epididymal white adipose tissue SVF snRNAseq	Sarvari et al, 2021https://github.com/JesperGrud/snRNAseq_eWAT	GSE160729
Inguinal white adipose tissue SnRNAseq	Emont et al, 2022https://gitlab.com/rosen-lab/white-adipose-atlas	GSE176171
Inguinal White Adipose tissue ScRNAseq of young and aged mice	Holman et al, 2024https://github.com/calhounr/Aging-impairs-cold-induced-beige-adipogenesis-and-adipocyte-metabolic-reprogramming	GSE227441
Inguinal WAT SVF from p12 and 10w mice ScRNAseq	Merrick et al, 2019	GSE128889

### Isolation and culturing of primary WAT and BAT progenitors

All animal work procedures were approved by the institutional animal care and use committee of the University of Wisconsin-Madison School of Medicine and Public Health and conducted in accordance with ethical regulations and policies. Primary WAT and BAT progenitors were isolated as previously described (Galmozzi et al, 2021). Briefly, subcutaneous white and interscapular brown depots were dissected from male and female C57/BL6 P1 mice into 250 μL ice-cold PBS and 200 μL 2X isolation buffer (123 mM NaCl, 5 mM KCl, 1.3 mM CaCl_2_, 5 mM glucose, 100 mM 4-(2-hydroxyethyl)-1-piperazineethanesulfonic acid (HEPES), penicillin–streptomycin, and 4% fatty acid-free bovine serum albumin). Tissues from four different animals were pooled, minced using small scissors, 50 μL of 15 mg/mL collagenase type I was added and immediately incubated at 37 °C on a shaker for 35 (WAT) and 50 (BAT) minutes, respectively. After digestion, samples were filtered through a 100 μm cell strainer into 10 mL isolation medium (Dulbecco’s modified Eagle medium (DMEM) + 20% fetal bovine serum (FBS), 10 mM HEPES, 1% penicillin–streptomycin) into a 10-cm dish and incubated at 37 °C, 5% CO_2_ for 1.5 h, followed by three washes with serum-free DMEM to remove blood cells and tissue debris. After washing, 10 mL of isolation medium was added, and cells were incubated overnight. The following day, cells were washed again with serum-free DMEM and maintained in isolation media, which were thereafter refreshed every 2 days. After reaching confluency (in 4 days), cells were washed with PBS and lifted with Trypsin-EDTA for 3 min. WAT and BAT progenitor suspensions were then washed in isolation media, counted, and live cells processed for single-cell sequencing.

### Single-cell RNA library preparation and sequencing

Single-cell RNA sequencing was conducted by the University of Wisconsin-Madison Biotechnology Center’s Gene Expression Center Core Facility using the 10x Genomics Chromium Single Cell 3′ v3.1 platform. Single-cell suspensions were loaded onto the Chromium Next GEM Chip, where cells were partitioned into Gel Bead-In-EMulsions (GEMs) for reverse transcription (RT). Post GEM-RT, cDNA was recovered and purified using DynaBeads MyOne Silane beads (Lot# 160221), followed by amplification through 12 cycles of PCR. The amplified cDNA underwent cleanup with SPRIselect beads (0.6X, Lot# 18198500), and quality was checked using an Agilent HS DNA chip. For library construction, cDNA was fragmented, end-repaired, and A-tailed, with double-sided size selection using SPRIselect beads (0.6x and 0.8x, Lot# 18198500). Adapter ligation was performed, followed by post-ligation cleanup with SPRIselect beads (0.8×). Index PCR was carried out with seven cycles using the Chromium Dual Index TT Set A (Lot# 160185). The final library was cleaned using SPRIselect beads (0.6× and 0.8×), recovering 35 μL in EB buffer. Library concentration was determined using a Qubit Fluorometer with Qubit dsDNA HS reagents. Final quality control was performed using an Agilent TapeStation with D1000 ScreenTape. Sequencing was initially conducted on a MiSeq Nano, followed by final sequencing on an SP flow cell.

### ScRNAseq data analysis

Single-cell RNA-seq data were analyzed by the UW Bioinformatics Resource Center and 10X Cloud services. Quality control of the MiSeq balancing run was performed using UMI-tools. Libraries were balanced for estimated reads per cell and sequenced on an Illumina NovaSeq system. Cell Ranger 7.0.1 software was utilized for demultiplexing, alignment, filtering, barcode counting, UMI counting, and gene expression estimation, using default parameters and the mouse mm10 2020-A genome. High-quality reads were obtained with over 93% mapping confidence. For the WAT sample, 5261 cells were captured with a mean of 48,966 reads per cell and a median of 5777 genes per cell. For the BAT sample, 7009 cells were captured with a mean of 48,249 reads per cell and a median of 5720 genes per cell. Investigators were not blinded to the experimental conditions.

Seurat v5 (Hao et al, 2024) was used to perform downstream filtering, log-normalization and analyses in R Studio. After applying filters for both WAT and BAT (3900>nFeature RNA > 7700, 1.7%< mitochondrial DNA < 5%, 6000< nCount RNA < 60,000) to remove low-quality reads, 4407 cells, 20,999 genes for WAT and 6173 cells, 21,889 genes for BAT were recovered. Next, non-adipo cells were removed from BAT and WAT, resulting in 4331 white precursors and 5958 brown precursors. Cell cycle scoring and regression were applied using default workflow parameters. Cluster analysis at 0.5 resolution with 10 dimensions calculated from the cell-cycle regressed PCA revealed the final clustering, and markers were identified using the Seurat FindMarkers function. Biological pathway analysis was performed with Metascape (Zhou et al, 2019) while molecular function was assigned using the PANTHER classification system (version 19.0).

Public datasets for WAT (Emont et al, 2022; Hepler et al, 2018; Holman et al, 2024; Merrick et al, 2019; Sarvari et al, 2021; Schwalie et al, 2018) and BAT (Burl et al, 2018; Karlina et al, 2021) adipocyte precursors were downloaded and re-processed using Seurat v5. Extraction of data was limited to preadipocytes control on a chow diet only. Clustering adjustments were made for the Merrick et al, 2019, Karlina et al, 2021, and Hepler et al, 2018 datasets to account for cross-platform differences and the availability of clustering parameters. In Hepler et al, dataset, due to the lack of available clustering parameters, the distinction between adipogenic cells (1A and 1B) was consolidated into a single unified APCs group. A clear distinction between APCs and FIPs was observed based on Ly6c1 expression, a key marker for FIPs (Hepler et al, 2018). Similarly, the Merrick and Karlina datasets were reanalyzed according to the corresponding published parameters using Seurat v5, resulting in highly consistent UMAP projections and clusters.

For reference mapping of public datasets for WAT and BAT against our datasets, common anchors were identified using the FindTransferAnchors function. This function aligns datasets by identifying shared cell features, facilitating integration of disparate datasets based on precomputed PCA space. Next, the MapQuery function was used to project the public datasets (query cells) onto our datasets’ UMAPs (reference cells), where 1246 anchors for Emont et al, 373 anchors for Hepler et al, 836 anchors for Sarvari et al, 442 anchors for Schwalie et al, 670 anchors for Holman et al, 498 (P12) and 652 (8 week-old) anchors for Merrick et al, 1199 anchors for Burl et al, and 265 anchors for Karlina et al were identified, respectively. The query cells, when mapped, were assigned a predicted identity based on their similarity to the reference clusters, alongside their original labels. The predicted labels were then quantified and represented as subcluster percentages. Finally, to illustrate the spatial alignment, the UMAP coordinates of WAT and BAT datasets with the mapped UMAP coordinates from the published datasets were merged.

For WAT and BAT aggregate, the WAT and BAT datasets were combined using the merge and reduce functions. After combining the two datasets, the cell cycle effect was regressed out of PCA.

### Pseudotime trajectory analysis

RNA velocity (Bergen et al, 2020) was constructed using the velocyto package, specific to the 10X platform, which generated an RNA velocity (.loom) file for both white and brown adipose tissue. Next, the UMAP coordinates and cluster identities from R/Seurat, together with the matrix reads and the newly generated .loom file, were used as inputs for ScVelo. Differentiation trajectories were analyzed and visualized using the top 2000 algorithmically defined driver genes using ScVelo’s dynamical modeling alongside Numpy, pandas, scanpy, anndata, igraph, loompy, and matplotlib, following the standard workflow. Fate probabilities were assessed via the CellRank2 workflow using the RNA velocity kernel (Lange et al, 2022) with time information extracted from ScVelo, and GPCCA (Reuter et al, 2019) (pyGPCCA package) for CellRank2 estimators input. The number of macrostates was chosen based on their robustness to parameter changes, and compute_lineage_drivers identified the driver genes of each lineage. To visualize the temporal bifurcation, the significant driver genes (filtered by log-*q* val < 0.05) for each lineage were extracted and their queried gene-expression models at each time point from cr.pl. heatmap were retrieved. Data were compiled into DataFrames and sorted in reverse order for one lineage. Temporal bifurcated expression data were visualized with pheatmap.

### Regulon scoring

Regulon activity was assessed using pySCENIC (Aibar et al, 2017), run within a Docker container for reproducibility. The SCENIC+ motif collection (Bravo Gonzalez-Blas et al, 2023) was obtained from resources.aertslab.org, alongside mm10 genomic region files centered on 500 bp and 10,000 bp around transcription start sites. Following the SCENIC protocol, pySCENIC first inferred the underlying gene regulatory network, then identified candidate regulons via RcisTarget motif enrichment analysis, and finally scored the activity of these regulons in each cell using the AUCell module. The resulting AUCell score matrix was then merged with the Seurat object for downstream analyses. Subsequent analyses were performed in Seurat and visualized with pheatmap, focusing on transcription factors whose activity patterns appeared especially robust or distinct across clusters through hierarchical clustering.

### Graphics

The models in Figs. 2 and 3 were created using BioRender.

## Supplementary information

Dataset EV1

Dataset EV2

Dataset EV3

Dataset EV4

Dataset EV5

Dataset EV6

Peer Review File

Source data Fig. 1

Source data Fig. 2

Source data Fig. 3

Source data Fig. 4

Source data Fig. 5

Expanded View Figures

## Data Availability

All data generated via scRNA-seq have been deposited in the Gene Expression Omnibus database under Gene Expression Omnibus accession number GSE304371. Processed RNA velocity analyses were deposited on Zenodo. Scripts used for the analyses were uploaded to GitHub at https://github.com/galmozzilab/mmAPC-subtypes. The source data of this paper are collected in the following database record: biostudies:S-SCDT-10_1038-S44319-025-00591-6.
