# Supplementary material for: White and brown adipose tissue share a convergent fibro-adipogenic progenitor population
Source: EMBO Rep. 2025 Oct 8;26(22):5612–36. doi: 10.1038/s44319-025-00591-6 (PMC12635263; doi:10.1038/s44319-025-00591-6)
Supplement: Supplementary file 13 — Expanded View Figures [file 44319_2025_591_MOESM13_ESM.pdf]

Expanded View Figures

Figure EV1. Cell cycle regression and re-clustering of WAT and BAT APCs.

(**A**) Clustering of WAT APCs before cell cycle regression. (**B**) Cell cycle phase of WAT APCs. (**C**) Distribution of cell cycle in WAT APC clusters from (**A**). (**D**) Cell cycle regression leads to redistribution of WAT APCs. (**E**) Cell cycle phases are equally distributed across clusters upon cell cycle regression. (**F**) Clustering of BAT APCs before cell cycle regression. (**G**) Cell cycle phase of BAT APCs. (**H**) Distribution of cell cycle in BAT APC clusters from (**F**). (**I**) Cell cycle regression leads to redistribution of BAT APCs. (**J**) Cell cycle phases are equally distributed across BAT clusters upon cell cycle regression.

© The Author(s)

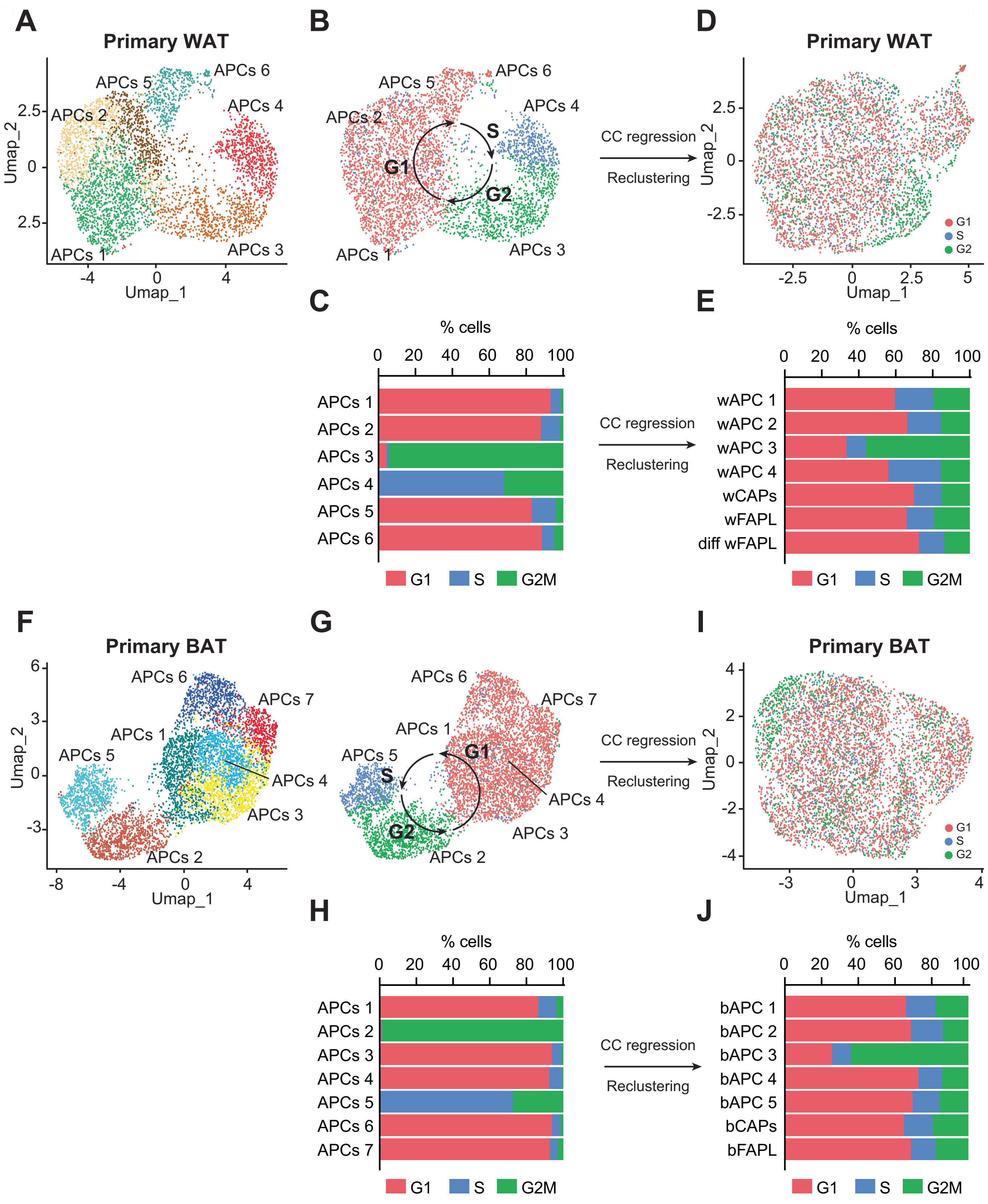
A
Primary WAT
APCs 1
APCs 2
APCs 3
APCs 4
APCs 5
APCs 6
Umap_1
Umap_2
B
G1
S
G2
CC regression
Reclustering
D
Primary WAT
G1
S
G2
C
% cells
0 20 40 60 80 100
G1 S G2M
E
wAPC 1
wAPC 2
wAPC 3
wAPC 4
wCAPs
wFAPL
diff wFAPL
F
Primary BAT
APCs 7
G
I
Primary BAT
H
J
bAPC 1
bAPC 2
bAPC 3
bAPC 4
bAPC 5
bCAPs
bFAPL

 © The Author(s)

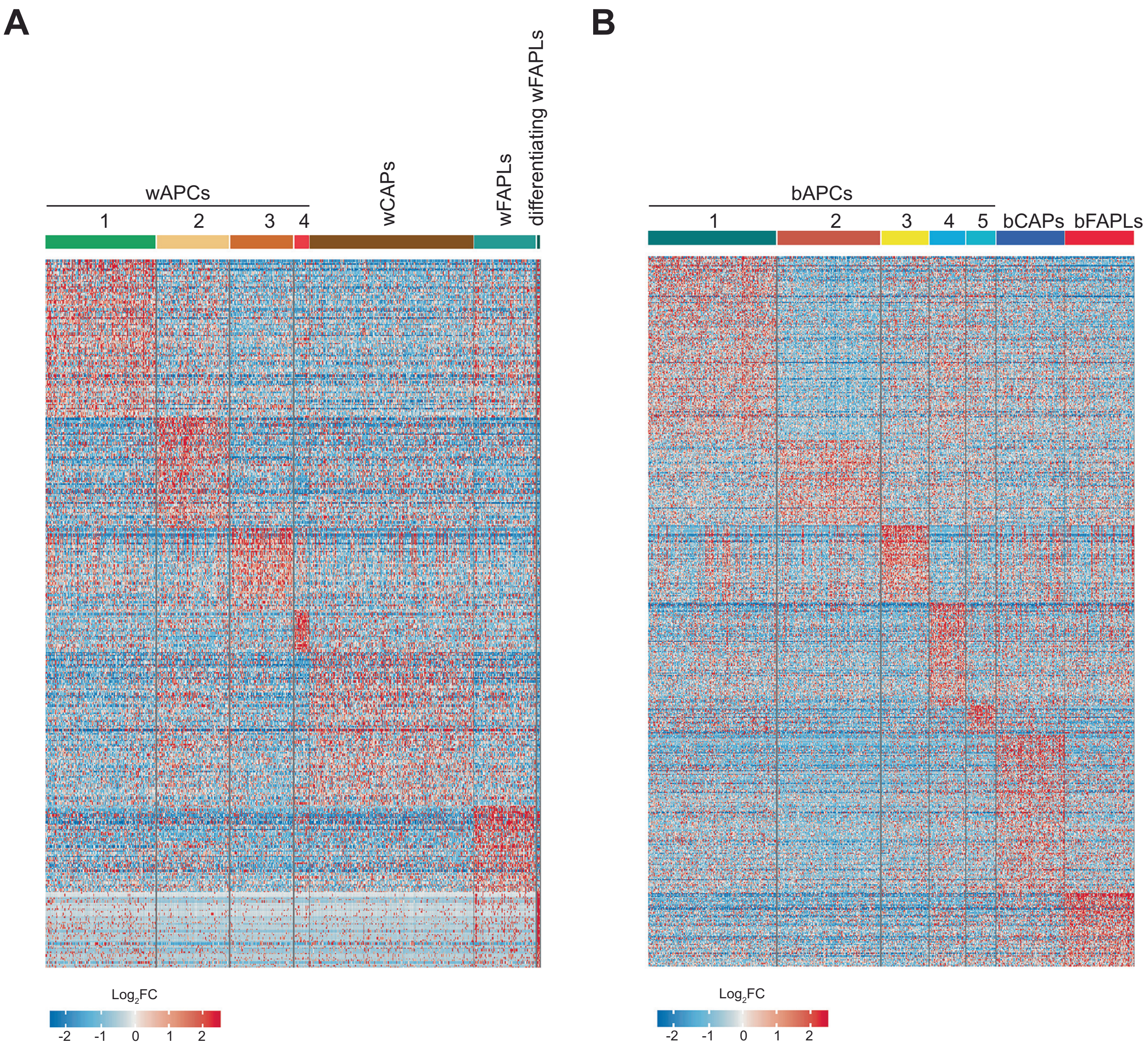

Figure EV2. WAT and BAT APC display unique transcriptional signatures.

(**A**) Heatmap of the top 10% differentially expressed genes for each white APC cluster. (**B**) Heatmap of the top 10% differentially expressed genes for each brown APC cluster.

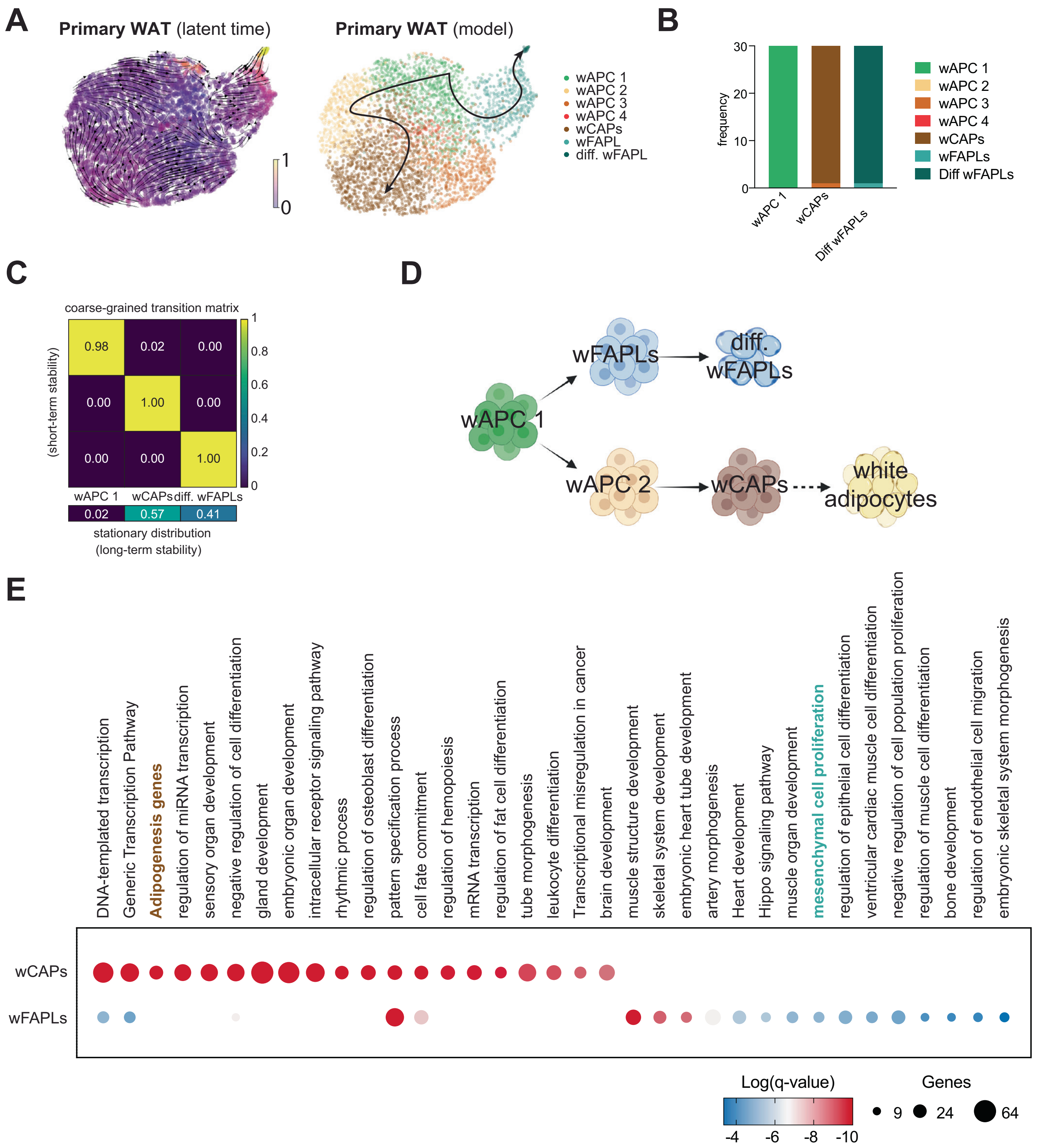

Figure EV3. Pseudotime trajectory inferences and macrostate assignments in WAT.

(**A**) Latent time UMAP visualization inferred by ScVelo and model of WAT APC differentiation trajectories highlight the transition from early precursors (wAPC 1) to wCAPs and wFAPLs. (**B**) Composition of WAT APC macrostates ($n = 30$ cells) by CellRank2. (**C**) Coarse-grained transition matrix using CellRank2 GPCCA for WAT macrostates indicating the stability (diagonal elements) and transition probabilities (stationary distribution) amongst macrostates. (**D**) Proposed developmental model of WAT APCs shows a shared early progenitor wAPC 1 that can differentiate into wFAPLs or wCAPs via intermediate transition states (wAPC 2). (**E**) Biological pathway analysis of the transcription factor drivers of wCAPs and wFAPLs shows enrichment in adipogenesis for wCAPs and mesenchymal-related pathways for wFAPLs.

 © The Author(s)

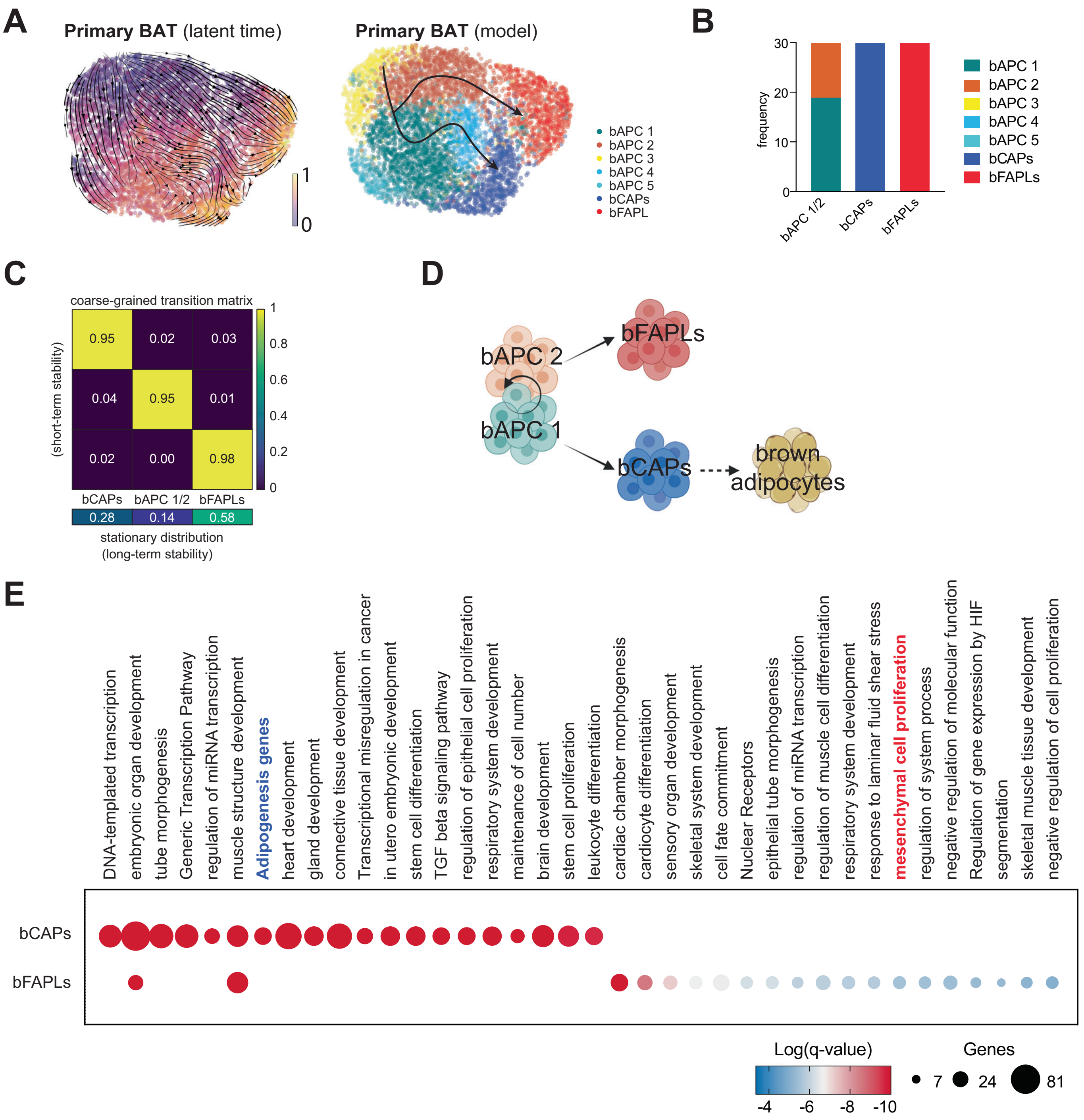

Figure EV4. Pseudotime trajectory inferences and macrostate assignments in BAT.

(**A**) Latent time UMAP visualization inferred by ScVelo and model of BAT APC differentiation trajectories highlight the transition from early precursors (bAPCs 1/2) to bCAPs and bFAPLs. (**B**) Composition of BAT APC macrostates ($n = 30$ cells) by CellRank2. (**C**) Coarse-grained transition matrix using CellRank2 GPCCA for WAT macrostates indicating the stability (diagonal elements) and transition probabilities (stationary distribution) amongst BAT macrostates. (**D**) Proposed developmental model of BAT APCs shows a mixed population of bAPC 1 and bAPC 2 that can differentiate into bCAPs and bFAPLs, respectively. (**E**) Biological pathway analysis of the transcription factor drivers of bCAPs and bFAPLs shows enrichment in adipogenesis for bCAPs and mesenchymal-related pathways for bFAPLs.

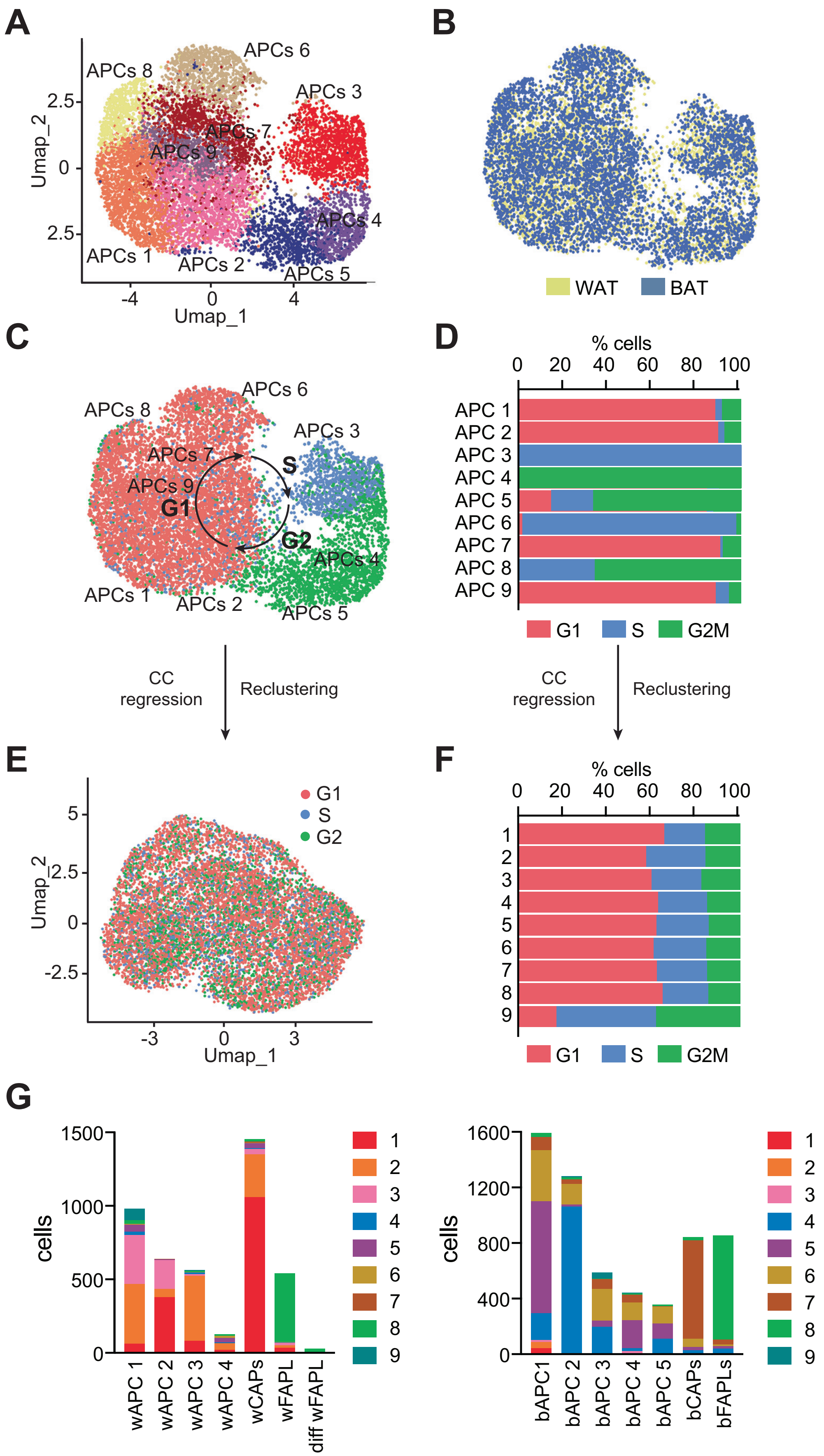
A
APCs 8
APCs 6
APCs 3
APCs 7
APCs 9
APCs 4
APCs 1
APCs 2
APCs 5
Umap_2
Umap_1
B
WAT
BAT
C
G1
S
G2
D
% cells
APC 1
APC 2
APC 3
APC 4
APC 5
APC 6
APC 7
APC 8
APC 9
G1
S
G2M
CC regression
Reclustering
E
F
G
cells
wAPC 1
wAPC 2
wAPC 3
wAPC 4
wCAPs
wFAPL
diff wFAPL
bAPC1
bAPC 2
bAPC 3
bAPC 4
bAPC 5
bCAPs
bFAPLs

Figure EV5. Integrated analysis of WAT and BAT precursor populations.

(**A**) Clustering of integrated WAT and BAT APCs before cell cycle regression. (**B**) Distribution of WAT and BAT APCs in the aggregate UMAP. (**C**, **D**) Cell cycle phase (G1, S, G2/M) of WAT and BAT APCs and distribution across clusters. (**E**) UMAP of WAT and BAT APC aggregate after cell cycle regression. (**F**) Quantification of cell cycle frequency across clusters. (**G**) Cell count of white and brown APCs across each combined cluster.

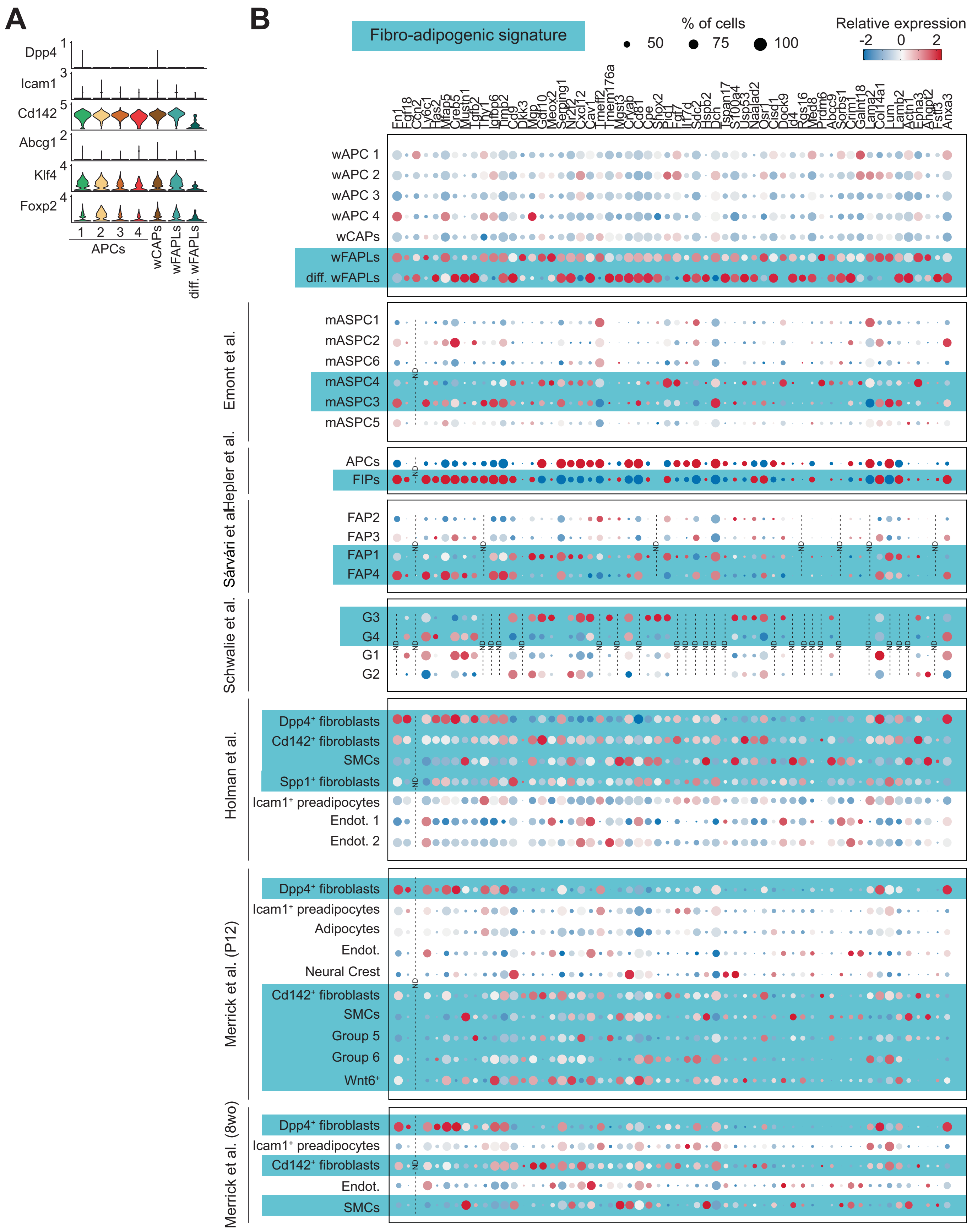
A
Dpp4
Icam1
Cd142
Abcg1
Klf4
Foxp2
1
3
5
2
4
4
1 2 3 4
APCs
wCAPs
wFAPLs
diff. wFAPLs
B
Fibro-adipogenic signature
% of cells
50
75
100
Relative expression
-2 0 2
En1
Fgf18
Ccn2
Ly6c1
Has2
Mfap5
Creb5
Mustn1
Tgfb2
Thy1
Igfbp6
Timp2
Cd9
Dkk3
Mgp
Gdf10
Meox2
Serping1
Nr2f2
Cxcl12
Cav1
Tmeff2
Tmem176a
Mgst3
Cryab
Cd81
Cpe
Shox2
Pid1
Fgf7
Il17d
Sdc2
Hspb2
Dcn
Tspan17
S100a4
Usp53
Naalad2
Osr1
Cisd1
Dock9
Id4
Rgs16
Med8
Prdm6
Abcc9
Sorbs1
Crim1
Galnt18
Lama2
Col14a1
Lum
Lamb2
Actn1
Epha3
Angpt2
Fstl3
Anxa3
wAPC 1
wAPC 2
wAPC 3
wAPC 4
wCAPs
wFAPLs
diff. wFAPLs
Emont et al.
mASPC1
mASPC2
mASPC6
mASPC4
mASPC3
mASPC5
Hepler et al.
APCs
FIPs
Sárvári et al.
FAP2
FAP3
FAP1
FAP4
Schwalie et al.
G3
G4
G1
G2
Holman et al.
Dpp4⁺ fibroblasts
Cd142⁺ fibroblasts
SMCs
Spp1⁺ fibroblasts
Icam1⁺ preadipocytes
Endot. 1
Endot. 2
Merrick et al. (P12)
Dpp4⁺ fibroblasts
Icam1⁺ preadipocytes
Adipocytes
Endot.
Neural Crest
Cd142⁺ fibroblasts
SMCs
Group 5
Group 6
Wnt6⁺
Merrick et al. (8wo)
Dpp4⁺ fibroblasts
Icam1⁺ preadipocytes
Cd142⁺ fibroblasts
Endot.
SMCs
ND

 © The Author(s)

Figure EV6. Identification of a shared FAP signature across multiple datasets.

(**A**) Expression in culture WAT APCs of known genes previously reported to define mASPC4, FIP, *Dpp4*$^{+}$, *Icam1*$^{+}$, *Cd142*$^{+}$, Aregs and FAP populations in the WAT APC datasets ($n = 4331$ cells). (**B**) Expression of other differentially expressed genes enriched in wFAPLs and differentiating wFAPLs compared to the expression found in previously reported fibroadipogenic populations.

© The Author(s)
