# Supplementary material for: White and brown adipose tissue share a convergent fibro-adipogenic progenitor population
Source: EMBO Rep. 2025 Oct 8;26(22):5612–36. doi: 10.1038/s44319-025-00591-6 (PMC12635263; doi:10.1038/s44319-025-00591-6)
Supplement: Supplementary file 7 — Peer Review File [file 44319_2025_591_MOESM7_ESM.pdf]

EMBO reports

White and Brown Adipose Tissue Share a Convergent Fibro-Adipogenic Progenitor Population

Hoang Bui, Julia Hansen, Valentina Lo Sardo, and Andrea Galmozzi

Corresponding author(s): Andrea Galmozzi (agalmozzi@medicine.wisc.edu)

Review Timeline:

Submission Date:	27th May 25
Editorial Decision:	25th Jul 25
Revision Received:	4th Aug 25
Editorial Decision:	30th Aug 25
Revision Received:	31st Aug 25
Accepted:	12th Sep 25

Editor: Deniz Senyilmaz Tiebe / Kurt Weir

Transaction Report:

(Note: With the exception of the correction of typographical or spelling errors that could be a source of ambiguity, letters and reports are not edited. Depending on transfer agreements, referee reports obtained elsewhere may or may not be included in this compilation. Referee reports are anonymous unless the Referee chooses to sign their reports.)

1st Editorial Decision 25th Jul 2025

Dear Andrea,

Thank you for submitting your manuscript to EMBO Reports, which was now seen by two referees, whose reports are copied below. My apologies for this unusual delay in getting back to you, it took longer than anticipated to receive the referee reports, as recently communicated.

Referees express interest in your single-cell RNA sequencing analysis revealing that your previously developed method (Galmozzi et al, 2021), which aimed to isolate white and brown preadipocytes from neonatal mice, succeeds in recapitulating the in vivo heterogeneity of adipocyte progenitors in vitro and identifying a cell population called fibro-adipogenic progenitors (FAPs) in the BAT. However, they, especially referee #1, also raise some concerns that need to be addressed to consider publication here.

I find the reports informed and constructive, and believe that addressing the concerns raised will significantly strengthen the manuscript. As the reports are below, and I think all points need to be addressed, I will not detail them here. Please contact me if you have questions or comments regarding the revision for further discussion (also by video chat).

Should you be able to address all referee concerns, we would like to invite you to revise your manuscript with the understanding that the referee concerns (as in their reports) must be fully addressed and their suggestions taken on board. Please address all referee concerns in a complete point-by-point response. Acceptance of the manuscript will depend on a positive outcome of a second round of review. It is EMBO reports policy to allow a single round of major experimental revision only and acceptance or rejection of the manuscript will therefore depend on the completeness of your responses included in the next, final version of the manuscript.

We realize that it is difficult to revise to a specific deadline. In the interest of protecting the conceptual advance provided by the work, we recommend a revision within 3 months. Please discuss the revision progress ahead of this time with me if you require more time to complete the revisions, or if you have questions or comments regarding the revision (also by video chat).

IMPORTANT NOTE: we perform an initial quality control of all revised manuscripts before re-review. Your manuscript will FAIL this control and the handling will be DELAYED if the following APPLIES:
1. A data availability section providing access to data deposited in public databases is missing (where applicable).
2. Your manuscript contains statistics and error bars based on n=2. Please use scatter plots in these cases.

You can submit the revision either as a Scientific Report or as a Research Article. For Scientific Reports, the revised manuscript can contain up to 5 main figures and 5 Expanded View figures, and it should not exceed 27000 characters. If the revision leads to a manuscript with more than 5 main figures it will be published as a Research Article. In this case the Results and Discussion section should be separate. If a Scientific Report is submitted, these sections have to be combined. This will help to shorten the manuscript text by eliminating some redundancy that is inevitable when discussing the same experiments twice. In either case, all materials and methods should be included in the main manuscript file.

When submitting your revised manuscript, please carefully review the instructions that follow below. Failure to include requested items will delay the evaluation of your revision.

1) a .docx formatted version of the manuscript text (including legends for main figures, EV figures and tables). Please make sure that the changes are highlighted to be clearly visible.

2) individual production quality figure files as .eps, .tif, .jpg (one file per figure). See https://wol-prod-cdn.literatumonline.com/pb-assets/embo-site/EMBOPress_Figure_Guidelines_061115-1561436025777.pdf for more info on how to prepare your figures.

3) We replaced Supplementary Information with Expanded View (EV) Figures and Tables that are collapsible/expandable online. A maximum of 5 EV Figures can be typeset. EV Figures should be cited as 'Figure EV1, Figure EV2" etc... in the text and their respective legends should be included in the main text after the legends of regular figures.

- For the figures that you do NOT wish to display as Expanded View figures, they should be bundled together with their legends in a single PDF file called *Appendix*, which should start with a short Table of Content. Appendix figures should be referred to in the main text as: "Appendix Figure S1, Appendix Figure S2" etc. See detailed instructions regarding expanded view here: <https://www.embopress.org/page/journal/14693178/authorguide#expandedview>

- Additional Tables/Datasets should be labeled and referred to as Table EV1, Dataset EV1, etc. Legends have to be provided in a separate tab in case of .xls files. Alternatively, the legend can be supplied as a separate text file (README) and zipped together with the Table/Dataset file.

4) a .docx formatted letter INCLUDING the reviewers' reports and your detailed point-by-point responses to their comments. As part of the EMBO publication's Transparent Editorial Process, EMBO reports publishes online a Review Process File (RPF) to accompany accepted manuscripts. This File will be published in conjunction with your paper and will include the referee reports, your point-by-point response and all pertinent correspondence relating to the manuscript.
https://www.embopress.org/page/journal/14693178/authorguide#transparentprocess
You are able to opt out of this by letting the editorial office know (emboreports@embo.org). If you do opt out, the Review Process File link will point to the following statement: "No Review Process File is available with this article, as the authors have chosen not to make the review process public in this case."

5) a complete author checklist, which you can download from our author guidelines https://www.embopress.org/page/journal/14693178/authorguide. Please insert information in the checklist that is also reflected in the manuscript. The completed author checklist will also be part of the RPF.

6) Please note that all corresponding authors are required to supply an ORCID ID for their name upon submission of a revised manuscript (<https://orcid.org/>). Please find instructions on how to link your ORCID ID to your account in our manuscript tracking system in our Author guidelines
<https://www.embopress.org/page/journal/14693178/authorguide#authorshipguidelines>

7) Before submitting your revision, primary datasets produced in this study need to be deposited in an appropriate public database (see https://www.embopress.org/page/journal/14693178/authorguide#datadeposition). Please remember to provide a reviewer password if the datasets are not yet public. The accession numbers and database should be listed in a formal "Data Availability" section placed after Materials & Method (see also https://www.embopress.org/page/journal/14693178/authorguide#datadeposition). Please note that the Data Availability Section is restricted to new primary data that are part of this study. * Note - All links should resolve to a page where the data can be accessed. *
If your study has not produced novel datasets, please mention this fact in the Data Availability Section.

8) At EMBO Press we ask authors to provide source data for the main manuscript figures. You will receive a separate email with instructions for providing source data with your revised manuscript, including how to upload and organize the files.

Additional information on source data and instruction on how to label the files are available:
https://www.embopress.org/page/journal/14693178/authorguide#sourcedata

9) Our journal encourages inclusion of *data citations in the reference list* to directly cite datasets that were re-used and obtained from public databases. Data citations in the article text are distinct from normal bibliographical citations and should directly link to the database records from which the data can be accessed. In the main text, data citations are formatted as follows: "Data ref: Smith et al, 2001" or "Data ref: NCBI Sequence Read Archive PRJNA342805, 2017". In the Reference list, data citations must be labeled with "[DATASET]". A data reference must provide the database name, accession number/identifiers and a resolvable link to the landing page from which the data can be accessed at the end of the reference. Further instructions are available at http://www.embopress.org/page/journal/14693178/authorguide#referencesformat

10) Regarding data quantification (see Figure Legends:
https://www.embopress.org/page/journal/14693178/authorguide#figureformat)

The following points must be specified in each figure legend:

- the name of the statistical test used to generate error bars and P values,

- the number (n) of independent experiments (please specify technical or biological replicates) underlying each data point,

- the nature of the bars and error bars (s.d., s.e.m.),

- If the data are obtained from n Program fragment delivered error ``Can't locate object method "less" via package "than" (perhaps you forgot to load "than"?) at //ejpvfs23/sites23b/embor_www/letters/embor_decision_revise_and_review.txt line 56.' 2, use scatter blots showing the individual data points.

Discussion of statistical methodology can be reported in the materials and methods section, but figure legends should contain a basic description of n, P and the test applied.

- Please also include scale bars in all microscopy images.

11) The journal requires a statement specifying whether or not authors have competing interests (defined as all potential or actual interests that could be perceived to influence the presentation or interpretation of an article). In case of competing interests, this must be specified in your disclosure statement. Further information: https://www.embopress.org/competing-interests

12) Please also note our reference format:
http://www.embopress.org/page/journal/14693178/authorguide#referencesformat

13) All Materials and Methods need to be described in the main text using our 'Structured Methods' format, which is required for all research articles. According to this format, the Methods section includes a Reagents and Tools Table (listing key reagents, experimental models, software and relevant equipment and including their sources and relevant identifiers) followed by a Methods and Protocols section describing the methods using a step-by-step protocol format. The aim is to facilitate adoption of the methodologies across labs. More information on how to adhere to this format as well as a downloadable template (.docx) for the Reagents and Tools Table can be found in our author guidelines:
https://www.embopress.org/page/journal/14693178/authorguide#structuredmethods.

An example of a Method paper with Structured Methods can be found here:
https://www.embopress.org/doi/10.15252/msb.20178071.

We would also welcome the submission of cover suggestions, or motifs to be used by our Graphics Illustrator in designing a cover.

I look forward to seeing a revised version of your manuscript when it is ready. Please let me know if you have questions or comments regarding the revision.

Kind regards,

Deniz

Deniz Senyilmaz Tiebe, PhD
Senior Scientific Editor
EMBO Reports

Referee #1:

The authors use single-cell RNA sequencing on primary white and brown preadipocytes. They identify two major populations, a committed adipogenic precursor and fibro-adipogenic progenitor-like cells. They find that fibro-adipogenic cells from white and brown fat behave similarly. These studies provide a comprehensive analysis of transcripts enriched in distinct populations. These studies provide some novel insights, while also aligning with previously published studies.

Referee #2:

The manuscript by Bui et al uses single-cell RNA sequencing to analyze cultured progenitors derived from both white and brown adipose tissues. The authors identify two key subpopulations committed adipogenic precursors (CAPs) and fibro-adipogenic progenitor-like cells (FAPLs) and convincingly demonstrate that FAPLs from both depots share conserved transcriptional programs, including stress-response and pro-fibrotic signatures, while CAPs remain depot-specific. By integrating their data with existing in vivo data they show that that subtypes of these in vitro cells closely resemble natural adipose progenitor subtypes, supporting the model's relevance for studying adipose tissue diversity and development. The data presented are of high quality, the analyses are quite comprehensive and understanding the differences and similarities between in vivo and in vitro as well as white and brown progenitor cell populations is important. Nevertheless, I have some concerns that I would like to discuss below.

Major concerns
1. A major point of this manuscript is the comparison between the cultured and in vivo preadipocytes that is presented in Figure 5. However, this comparison is confounded by two axes of variation, the difference between in vitro and in vivo cells, and the difference between neonatal and adult mice. This raises concerns about direct comparability, particularly since APC clusters 1-5 in your dataset do not align well with previously defined in vivo populations. Mapping to a dataset such as PMID: 31023895 which performed single cell RNA-seq on p10 pups might make the comparison clearer, as would mapping to datasets that performed single cell or single nuclear RNA sequencing on cultured preadipocytes derived from adult animals, if these data are available.

2. Related to the above, while the discussion of Figure 5 indicates that "Cultured APCs recapitulate the heterogeneity of APCs in vivo", the mapping suggests that there are a number of APC populations that are seen in vitro and not in vivo, specifically wAPC1, wAPC3, and wAPC4. What is your assessment of these populations and why they are not seen in vivo? Do you believe age is a significant factor affecting progenitor identity or differentiation capacity? Clarifying this point will strengthen the relevance of your model.
3. While the title suggests a common fibro-adipogenic progenitor population in white and brown adipose tissues, there are clearly still differences, for example Figures 1H and 1I show distinct gene expression profiles between wFAPLs and bFAPLs and Figure 4F shows that, while there are more shared genes compared to CAPs, there are still differential genes in FAPLs. Additionally, previous work such as PMID: 18719582 specifically points to distinct developmental origins of brown and white preadipocytes. How do you reconcile this?
4. Similar to the above, how was the comparison of marker genes in Figure 4F performed? If possible and applicable, I would recommend using pseudobulk analysis for this type of comparison as it results in fewer false discoveries than methods that find markers between conditions in the single cell data (see PMID: 34584091).
5. In Figure 2C, several CAP trajectory genes, such as Prdm16, Ebf2, and Ppargc1a which are more typically associated with brown/beige adipocyte biology are seen in the white CAPs but not the brown ones in Figure 3C. Could you expand on this discrepancy?
6. According to Figure 2A, wAPC1 gives rise to both wCAPs and wFAPLs, yet Figure 2F highlights regulon scores for wAPC2-wCAPs and not wAPC1. This is somewhat confusing. Please clarify this.
7. While Figure 4D presents marker expression for cluster 8, which includes both WAT and BAT cells, cluster 9 is also described as a mixed population. Would it be possible to include a similar marker analysis for cluster 9?
Minor concerns
1. The results section does not call out Figure 2D. Please describe this figure in the results section.
2. Please clarify the identity of the "oligod" cluster in Figures 1D and 1E.
3. It would be helpful to highlight Ucp1 expression more explicitly in Figure 1I, as this could offer additional insights into the functional potential of the identified populations.

RESPONSE TO REFEREES – EMBOR-2025-62003V1

We thank the Reviewers for their interest in our work and for their careful and insightful comments. We have incorporated their suggestions, which have further strengthened our manuscript. As per the Editor's guidelines, we have modified our manuscript to meet the formatting requirements of a Research Article that includes **5 main** and **6 Extended View figures**. We also edited the text to incorporate the new results. During this process, we carefully considered all the text-related suggestions from the Reviewers. A point-by-point response to their specific comments follows.

Referee #1

The authors use single-cell RNA sequencing on primary white and brown preadipocytes. They identify two major populations, a committed adipogenic precursor and fibro-adipogenic progenitor-like cells. They find that fibro-adipogenic cells from white and brown fat behave similarly. These studies provide a comprehensive analysis of transcripts enriched in distinct populations. These studies provide some novel insights, while also aligning with previously published studies.

We thank Referee 1 for Their positive assessment and for recognizing the novelty and relevance of our study. We appreciate Their notes on our approach, the identification of distinct preadipocyte populations, and the transcriptomic similarities between fibro-adipogenic cells from white and brown fat. We are glad They found that our findings offer new insights while reinforcing existing literature.

Referee #2

The manuscript by Bui et al uses single-cell RNA sequencing to analyze cultured progenitors derived from both white and brown adipose tissues. The authors identify two key subpopulations committed adipogenic precursors (CAPs) and fibro-adipogenic progenitor-like cells (FAPLs) and convincingly demonstrate that FAPLs from both depots share conserved transcriptional programs, including stress-response and pro-fibrotic signatures, while CAPs remain depot-specific. By integrating their data with existing in vivo data they show that that subtypes of these in vitro cells closely resemble natural adipose progenitor subtypes, supporting the model's relevance for studying adipose tissue diversity and development. The data presented are of high quality, the analyses are quite comprehensive and understanding the differences and similarities between in vivo and in vitro as well as white and brown progenitor cell populations is important. Nevertheless, I have some concerns that I would like to discuss below.

We thank Referee 2 for Their thorough review and thoughtful comments on our manuscript. We are pleased that They found the data quality high and the analysis comprehensive, and we appreciate Their recognition of the relevance of our findings to understanding adipose progenitor heterogeneity. We have carefully considered Their concerns and provide detailed responses to each point below.

Major concerns

1. A major point of this manuscript is the comparison between the cultured and in vivo preadipocytes that is presented in Figure 5. However, this comparison is confounded by two axes of variation, the difference between in vitro and in vivo cells, and the difference between neonatal and adult mice. This raises concerns about direct comparability, particularly since APC clusters 1-5 in your dataset do not align well with previously defined in vivo populations. Mapping to a dataset such as PMID: 31023895 which performed single cell RNA-seq on p10 pups might make the comparison clearer, as would mapping to datasets that performed single

cell or single nuclear RNA sequencing on cultured preadipocytes derived from adult animals, if these data are available.

We thank the Referee for this insightful and important comment. We agree that both the *in vitro* vs. *in vivo* context and the developmental stage of the animals are key variables when interpreting comparisons across datasets. As suggested, we have now incorporated the two scRNA-seq datasets from Merrick et al., 2019 (PMID: 31023895) into our analysis (**Figure 5**). Notably, Merrick and colleagues profiled stromal cells from both postnatal day 12 (P12) pups and young adult (8-week-old) mice, offering a useful perspective on age-related changes.

As the Referee correctly points out, the developmental stage strongly influences adipose progenitor composition and their mapping onto our dataset. When integrating our data with the P12 dataset, we observed that *Dpp4*$^{+}$ fibroblasts representing early adipocyte progenitors mapped most closely to our wAPC 1 cluster, which we defined as a multipotent and least committed progenitor population. In contrast, when mapped to the 8-week-old dataset, *Dpp4*$^{+}$ cells predominantly aligned with our FAPL population, indicating a shift toward a more differentiated state. These results suggest that early-stage progenitors like wAPC 1 are enriched in neonatal tissues and gradually transition into intermediate states, such as FAPLs, as animals grow. This developmental progression is further supported by our integration with the dataset from Hepler et al. (PMID: 30265241), which used 6-week-old mice. In that case, FIPs mapped to both wAPC 1 and FAPL clusters, consistent with an intermediate developmental stage. By 8 weeks of age, which marks the onset of adulthood, wAPC 1 cells were virtually absent, and the dominant populations observed were CAPs and FAPLs, paralleling the mapping results obtained when comparing other datasets from older adult mice.

Finally, we agree that direct comparison with cultured preadipocytes derived from adult animals would be highly valuable. However, to the best of our knowledge, no such single-cell or single-nucleus RNA-seq dataset currently exists. While such comparisons would need to account for differences in isolation procedures, culture conditions, and the inherently lower proliferation capacity of adult progenitors, we would anticipate a similar trend: a loss of early, multipotent populations (e.g., wAPC 1 and possibly wAPC 2) and a relative enrichment of more committed states such as CAPs and FAPLs.

2. Related to the above, while the discussion of Figure 5 indicates that "Cultured APCs recapitulate the heterogeneity of APCs in vivo", the mapping suggests that there are a number of APC populations that are seen in vitro and not in vivo, specifically wAPC1, wAPC3, and wAPC4. What is your assessment of these populations and why they are not seen in vivo? Do you believe age is a significant factor affecting progenitor identity or differentiation capacity? Clarifying this point will strengthen the relevance of your model.

We appreciate the Referee's thoughtful observation regarding the presence of certain APC populations, specifically wAPC 1, wAPC 3, and wAPC 4, *in vitro* but not in the *in vivo* datasets included in our comparison. As discussed above, we believe age is a critical factor that influences the detection of early progenitor states such as wAPC 1. This population aligns closely with *Dpp4*$^{+}$ fibroblasts from postnatal (P12) mice but becomes progressively less prominent in datasets derived from older animals. This suggests that multipotent progenitor populations like wAPC 1 may be enriched in neonatal adipose tissue and gradually transition into more committed states as development proceeds. Thus, their apparent absence in adult *in vivo* datasets may reflect true biological maturation rather than technical or analytical limitations.

In contrast, a different explanation may account for the absence of wAPC3 and wAPC4 from *in vivo* datasets. Our trajectory and metastability analyses (**Figure 2** and **Figure EV3**) suggest that these populations likely represent transitional or intermediate stages rather than stable, distinct progenitor cell states. *In vitro* systems, by virtue of reduced cellular and environmental complexity compared to *in vivo* tissue, may provide a clearer window into such transient phases of cell state

transitions. The relative uniformity of the cultured conditions likely amplifies these intermediate stages, making them more detectable than in the heterogeneous and dynamic *in vivo* environment, where such transitions may be brief or masked by more pronounced differences among tissue-resident cell types.

Together, these observations emphasize two key points: (1) the developmental stage of the tissue plays a significant role in determining progenitor identity and composition, and (2) *in vitro* culture can reveal transitional cell states that may be difficult to resolve *in vivo*. Rather than diminishing the relevance of our model, we believe this underscores its utility for dissecting progenitor state dynamics and clarifying lineage progression within adipose tissue depots.

3. While the title suggests a common fibro-adipogenic progenitor population in white and brown adipose tissues, there are clearly still differences, for example Figures 1H and 1I show distinct gene expression profiles between wFAPLs and bFAPLs and Figure 4F shows that, while there are more shared genes compared to CAPs, there are still differential genes in FAPLs. Additionally, previous work such as PMID: 18719582 specifically points to distinct developmental origins of brown and white preadipocytes. How do you reconcile this?

We thank the Referee for raising this important point. We fully agree that white and brown preadipocytes have distinct developmental origins and do not arise from a shared progenitor. Our own data support this distinction. As shown in Figure 4E, white and brown FAPLs, while clustering together in the integrated analysis, originate from separate lineage-specific progenitors: wFAPLs from *Hoxc10*$^+$/*Hoxc9*$^+$ cells and bFAPLs from *Zic1*$^+$ cells. Therefore, our findings do not contradict known developmental differences; rather, they highlight a convergence in gene expression and functional characteristics at the level of FAPL cells. Despite arising from distinct origins, wFAPLs and bFAPLs exhibit remarkably similar molecular profiles, including consistent enrichment in stress-response and pro-fibrotic gene programs (**Figures 1H–I**, **2E**, **3E**, and **4F**). We interpret this as evidence of transcriptional and functional convergence, rather than shared developmental origin. To more accurately reflect this distinction and avoid ambiguity, we have revised the manuscript accordingly. We have also updated the title from *"White and Brown Adipose Tissue Share a Common Fibro-Adipogenic Progenitor Population"* to *"White and Brown Adipose Tissue Share a Convergent Fibro-Adipogenic Progenitor Population."*

4. Similar to the above, how was the comparison of marker genes in Figure 4F performed? If possible and applicable, I would recommend using pseudobulk analysis for this type of comparison as it results in fewer false discoveries than methods that find markers between conditions in the single cell data (see PMID: 34584091).

We thank the Referee for this helpful suggestion and have expanded the description of the analysis underlying **Figure 4F**. Briefly, differentially expressed genes (DEGs) were identified by comparing wFAPLs (or wCAPs) versus all other cells within the WAT dataset, and bFAPLs (or bCAPs) versus all other cells within the BAT dataset. We acknowledge the limitations of single-cell differential expression analysis, particularly the elevated risk of false discoveries when cells are treated as independent observations and differences arise from small subsets of highly-expressing cells within clusters. As the Referee noted, pseudobulk analysis is a robust alternative that reduces false discovery rates by accounting for biological variability across replicates (PMID: 34584091). However, in our study, cells from four independent neonatal mice were pooled prior to library preparation and sequencing for both the WAT and BAT datasets. As a result, only one sequencing library was generated per depot, precluding the use of true biological replicates required for pseudobulk analysis. Pseudo-replicates do not accurately capture biological variability and, therefore, do not provide a reliable basis for pseudobulk-based inference (PMID: 34584091). Consistent with this, pseudobulk analysis performed using three pseudo-replicates of

our datasets identified considerably more DEGs for FAPLs and CAPs in both WAT and BAT.

Figure for referee with unpublished data and its description has been removed upon request by the authors.

Nevertheless, to overcome this limitation and increase confidence in the identified DEGs, we performed an additional analysis to assess the robustness of gene expression across cells. Specifically, for the list of DEGs shared between white and brown FAPLs (and between CAPs), we calculated the percentage of cells within each group that expressed each gene. We then applied a stringent threshold, retaining only genes expressed in at least 75% of cells in both depots. This approach allowed us to filter out DEGs potentially driven by a small number of high-expressing cells. Notably, we found that the vast majority of DEGs shared by WAT and BAT clusters met this criterion, indicating broad and consistent expression across both white and brown FAPLs or CAPs. These results have been incorporated into a new **Figure 4F**.
While we recognize that analytical limitations remain, this additional filtering step strengthens the evidence that the identified DEGs represent *bona fide* markers of FAPL and CAP populations, independent of depot origin.

5. In Figure 2C, several CAP trajectory genes, such as Prdm16, Ebf2, and Ppargc1a which are more typically associated with brown/beige adipocyte biology are seen in the white CAPs but not the brown ones in Figure 3C. Could you expand on this discrepancy?
We thank the Referee for pointing this out and apologize for the confusion caused by the omission. Classical transcription factors and regulators associated with brown adipocyte differentiation, such as Prdm16, Ebf2, and Ppargc1a, were indeed identified among the top driver genes for bCAPs, as reported in the complete list of driver genes in **Table EV4**. These genes were not highlighted in Figure 3C, which may have contributed to the apparent discrepancy. We have now revised **Figure 3C** to clearly indicate the presence of these key regulators in the bCAP trajectory, ensuring consistency with the data presented in **Figure 2C**.

6. According to Figure 2A, wAPC1 gives rise to both wCAPs and wFAPLs, yet Figure 2F highlights regulon scores for wAPC2-wCAPs and not wAPC1. This is somewhat confusing. Please clarify this.

We appreciate the Referee's observation and are happy to clarify. In our trajectory analysis, wAPC 2 appears to function as a transitional intermediate between wAPC 1 and wCAPs, which is why we focused on the wAPC 2 - wCAP axis in the regulon analysis shown in **Figure 2F**. In contrast, the trajectory from wAPC 1 to wFAPLs does not involve an intermediate state, and wAPC 1 cells are more transcriptionally aligned with wFAPLs. Accordingly, in the regulon analysis, wAPC 1 clusters closer to wFAPLs, reflecting its more direct differentiation path toward that lineage. We have revised the text to clarify this point.

7. While Figure 4D presents marker expression for cluster 8, which includes both WAT and BAT cells, cluster 9 is also described as a mixed population. Would it be possible to include a similar marker analysis for cluster 9?

As requested, we have included representative markers of cluster 9 in **Figure 4D**.

Minor concerns

1. The results section does not call out Figure 2D. Please describe this figure in the results section.

We apologize for the oversight. We have revised the text to explicitly reference and describe **Figure 2D** in the appropriate section.

2. Please clarify the identity of the "oligod" cluster in Figures 1D and 1E.

We have updated **Figure 1** to ensure consistency in cluster naming. The "Oligod." clusters are now correctly labeled as "glial cells".

3. It would be helpful to highlight Ucp1 expression more explicitly in Figure 1I, as this could offer additional insights into the functional potential of the identified populations.

Ucp1 was not included in the panel of cluster-specific markers because it was not detected in any of the brown progenitor cells analyzed.

1st Revision - Editorial Decision 30th Aug 2025

Dear Andrea,

Thank you for submitting your revised manuscript. It has now been seen by one of the original referees.

As you will see, referee finds that the study is significantly improved during revision and recommend publication. However, the editorial points below need to be addressed before I can accept the manuscript.

• Please provide 3-5 keywords for your study. These will be visible in the html version of the paper and on PubMed and will help increase the discoverability of your work.
• We note the following regarding the funding information, which should be complete in both the manuscript text and the manuscript tracking system: missing in the manuscript tracking system: the Department of Medicine Pilot Funding Program at the University of Wisconsin-Madison School of Medicine and Public Health; grant number in the manuscript tracking system should be corrected to 1R35GM150899 instead of R35GM150899.
• Please remove the Author Contribution section from the manuscript text.
• Source file names, titles, legends and manuscript callouts all need to be updated to Dataset EV1-EV# instead of Tables EV1-EV6, legends should be removed from manuscript and only kept as a separate tab/sheet in each Excel file.
• Appendix file needs to be submitted in pdf format.
• All research articles submitted as revised versions must include a structured methods section that includes a Reagents and Tools Table followed by a Methods and Protocols section. This file should be submitted as a separate word file, not as a part of the manuscript text. Please see https://www.embopress.org/page/journal/14693178/authorguide#structuredmethods for further information.
• Please make the dataset GSE304371 publicly available and add a URL, that directly resolves to the dataset, into the Data Availability section.
• Similarly, please make sure that zenodo dataset is publicly accessible and add a URL, that directly resolves to the dataset, into the Data Availability section.
• Sections need to be named and the order should be corrected: Title page - Abstract - Keywords - Introduction - Results - Discussion - Methods - Data Availability - Acknowledgements - Disclosure and Competing Interests Statement - References - Figure Legends - Table(s) - Expanded View Figure Legends.
• Our production/data editors have asked you to clarify several points in the figure legends - Figure Legends (main + EV):
o Figure Legends (main + EV): 1. Please note that information related to n is missing in the legends of figures 1D, E H, I; 4D, E; EV6 A.
• Papers published in EMBO Reports include a 'synopsis' and 'bullet points' to further enhance discoverability. Both are displayed on the html version of the paper and are freely accessible to all readers. The synopsis includes a short standfirst summarizing the study in 1 or 2 sentences (max 35 words) that summarize the paper and are provided by the authors and streamlined by the handling editor. I would therefore ask you to include your synopsis blurb and 3-5 bullet points listing the key experimental findings.
• In addition, please provide an image for the synopsis. This image should provide a rapid overview of the question addressed in the study but still needs to be kept fairly modest since the image size cannot exceed 550 (width) x 300-600 (height) pixels.

Thank you again for giving us to consider your manuscript for EMBO Reports, I look forward to your minor revision.

Kind regards,

Deniz

--
Deniz Senyilmaz Tiebe, PhD
Senior Scientific Editor
EMBO Reports

Referee #2:

The authors have satisfactorily responded to my concerns.

All editorial and formatting issues were resolved by the authors.

Dr. Andrea Galmozzi
University of Wisconsin School of Medicine and Public Health
Medicine
United States

Dear Dr. Galmozzi,

I am very pleased to accept your manuscript for publication in the next available issue of EMBO reports. Thank you for your contribution to our journal.

Your manuscript will be processed for publication by EMBO Press. It will be copy edited and you will receive page proofs prior to publication. Please note that you will be contacted by Springer Nature Author Services to complete licensing and payment information.

You may qualify for financial assistance for your publication charges - either via a Springer Nature fully open access agreement or an EMBO initiative. Check your eligibility: https://www.embopress.org/page/journal/14693178/authorguide#chargesguide

Should you be planning a Press Release on your article, please get in contact with embo_production@springernature.com as early as possible in order to coordinate publication and release dates.

If you have any questions, please do not hesitate to contact the Editorial Office. Thank you for your contribution to EMBO Reports.

Yours sincerely,

Kurt Weir
Editor
EMBO Reports

>>> Please note that it is EMBO Reports policy for the transcript of the editorial process (containing referee reports and your response letter) to be published as an online supplement to each paper. If you do NOT want this, you will need to inform the Editorial Office via email immediately. More information is available here: https://www.embopress.org/transparent-process#Review_Process

EMBO Press Author Checklist

Corresponding Author Name: Andrea Galmozzi
Journal Submitted to: EMBO Reports
Manuscript Number: EMBOR-2025-62003V1

USEFUL LINKS FOR COMPLETING THIS FORM
The EMBO Journal - Author Guidelines
EMBO Reports - Author Guidelines
Molecular Systems Biology - Author Guidelines
EMBO Molecular Medicine - Author Guidelines

Reporting Checklist for Life Science Articles (updated January
This checklist is adapted from Materials Design Analysis Reporting (MDAR) Checklist for Authors. MDAR establishes a minimum set of requirements in transparent reporting in the life sciences (see Statement of Task: 10.31222/osf.io/9sm4x). Please follow the journal's guidelines in preparing your
Please note that a copy of this checklist will be published alongside your article.

Abridged guidelines for figures
1. Data
The data shown in figures should satisfy the following conditions:
- the data were obtained and processed according to the field's best practice and are presented to reflect the results of the experiments in an accurate and unbiased manner.
- ideally, figure panels should include only measurements that are directly comparable to each other and obtained with the same assay.
- plots include clearly labeled error bars for independent experiments and sample sizes. Unless justified, error bars should not be shown for technical
- if n<5, the individual data points from each experiment should be plotted. Any statistical test employed should be justified.
- Source Data should be included to report the data underlying figures according to the guidelines set out in the authorship guidelines on Data

2. Captions
Each figure caption should contain the following information, for each panel where they are relevant:
- a specification of the experimental system investigated (eg cell line, species name).
- the assay(s) and method(s) used to carry out the reported observations and measurements.
- an explicit mention of the biological and chemical entity(ies) that are being measured.
- an explicit mention of the biological and chemical entity(ies) that are altered/varied/perturbed in a controlled manner.
- the exact sample size (n) for each experimental group/condition, given as a number, not a range;
- a description of the sample collection allowing the reader to understand whether the samples represent technical or biological replicates (including how many animals, litters, cultures, etc.).
- a statement of how many times the experiment shown was independently replicated in the laboratory.
- definitions of statistical methods and measures:
  - common tests, such as t-test (please specify whether paired vs. unpaired), simple χ2 tests, Wilcoxon and Mann-Whitney tests, can be unambiguously identified by name only, but more complex techniques should be described in the methods section;
  - are tests one-sided or two-sided?
  - are there adjustments for multiple comparisons?
  - exact statistical test results, e.g., P values = x but not P values < x;
  - definition of 'center values' as median or average;
  - definition of error bars as s.d. or s.e.m.

Please complete ALL of the questions below.
Select "Not Applicable" only when the requested information is not relevant for your study.

Materials

Newly Created Materials	Information included in the manuscript?	In which section is the information available? (Reagents and Tools Table, Materials and Methods, Figures, Data Availability Section)
New materials and reagents need to be available; do any restrictions apply?	Not Applicable	

Antibodies	Information included in the manuscript?	In which section is the information available? (Reagents and Tools Table, Materials and Methods, Figures, Data Availability Section)
For **antibodies** provide the following information:<br>- Commercial antibodies: RRID (if possible) or supplier name, catalogue number and or/clone number<br>- Non-commercial: RRID or citation	Not Applicable	

DNA and RNA sequences	Information included in the manuscript?	In which section is the information available? (Reagents and Tools Table, Materials and Methods, Figures, Data Availability Section)
**Short novel DNA or RNA including primers, probes**: provide the sequences.	Not Applicable	

Cell materials	Information included in the manuscript?	In which section is the information available? (Reagents and Tools Table, Materials and Methods, Figures, Data Availability Section)
**Cell lines:** Provide species information, strain. Provide accession number in repository **OR** supplier name, catalog number, clone number, and/**OR** RRID.	Not Applicable	
**Primary cultures:** Provide species, strain, sex of origin, genetic modification status.	Yes	Methods
Report if the cell lines were recently **authenticated** (e.g., by STR profiling) and tested for mycoplasma contamination.	Not Applicable	

Experimental animals	Information included in the manuscript?	In which section is the information available? (Reagents and Tools Table, Materials and Methods, Figures, Data Availability Section)
**Laboratory animals or Model organisms:** Provide species, strain, sex, age, genetic modification status. Provide accession number in repository **OR** supplier name, catalog number, clone number, **OR** RRID.	Not Applicable	
**Animal observed in or captured from the field:** Provide species, sex, and age where possible.	Not Applicable	
Please detail **housing and husbandry conditions**.	Not Applicable	

Plants and microbes	Information included in the manuscript?	In which section is the information available? (Reagents and Tools Table, Materials and Methods, Figures, Data Availability Section)
**Plants:** provide species and strain, ecotype and cultivar where relevant, unique accession number if available, and source (including location for collected wild specimens).	Not Applicable	
**Microbes:** provide species and strain, unique accession number if available, and source.	Not Applicable	

Human research participants	Information included in the manuscript?	In which section is the information available? (Reagents and Tools Table, Materials and Methods, Figures, Data Availability Section)
If collected and within the bounds of privacy constraints report on age, sex and gender or ethnicity for all study participants.	Not Applicable	

Core facilities	Information included in the manuscript?	In which section is the information available? (Reagents and Tools Table, Materials and Methods, Figures, Data Availability Section)
If your work benefited from core facilities, was their service mentioned in the acknowledgments section?	Yes	Acknowledgments

Design

Study protocol	Information included in the manuscript?	In which section is the information available? (Reagents and Tools Table, Materials and Methods, Figures, Data Availability Section)
If study protocol has been **pre-registered, provide DOI in the manuscript**. For clinical trials, provide the trial registration number **OR** cite DOI.	Not Applicable	
Report the **clinical trial registration number** (at ClinicalTrials.gov or equivalent), where applicable.	Not Applicable	

Laboratory protocol	Information included in the manuscript?	In which section is the information available? (Reagents and Tools Table, Materials and Methods, Figures, Data Availability Section)
Provide DOI OR other citation details if **external detailed step-by-step protocols** are available.	Yes	Methods and references

Experimental study design and statistics	Information included in the manuscript?	In which section is the information available? (Reagents and Tools Table, Materials and Methods, Figures, Data Availability Section)
Include a statement about **sample size** estimate even if no statistical methods were used.	Yes	Methods
Were any steps taken to minimize the effects of subjective bias when allocating animals/samples to treatment (e.g. **randomization procedure**)? If yes, have they been described?	Not Applicable	
Include a statement about **blinding** even if no blinding was done.	Yes	Methods
Describe **inclusion/exclusion criteria** if samples or animals were excluded from the analysis. Were the criteria pre-established?<br><br>If sample or data points were omitted from analysis, report if this was due to attrition or intentional exclusion and provide justification.	Not Applicable	
For every figure, are **statistical tests** justified as appropriate? Do the data meet the assumptions of the tests (e.g., normal distribution)? Describe any methods used to assess it. Is there an estimate of variation within each group of data? Is the variance similar between the groups that are being statistically compared?	Yes	Methods and Data Availability

Sample definition and in-laboratory replication	Information included in the manuscript?	In which section is the information available? (Reagents and Tools Table, Materials and Methods, Figures, Data Availability Section)
In the figure legends: state number of times the experiment was **replicated** in laboratory.	Not Applicable	
In the figure legends: define whether data describe **technical or biological replicates**.	Not Applicable	

Ethics

Ethics	Information included in the manuscript?	In which section is the information available? (Reagents and Tools Table, Materials and Methods, Figures, Data Availability Section)
Studies involving **human participants**: State details of **authority granting ethics approval** (IRB or equivalent committee(s), provide reference number for approval.	Not Applicable	
Studies involving **human participants**: Include a statement confirming that **informed consent** was obtained from all subjects and that the experiments conformed to the principles set out in the WMA Declaration of Helsinki and the Department of Health and Human Services Belmont Report.	Not Applicable	
Studies involving **human participants:** For publication of **patient photos**, include a statement confirming that consent to publish was obtained.	Not Applicable	
Studies involving experimental **animals**: State details of **authority granting ethics approval** (IRB or equivalent committee(s), provide reference number for approval. Include a statement of compliance with ethical regulations.	Yes	Methods
Studies involving **specimen and field samples:** State if relevant **permits** obtained, provide details of authority approving study; if none were required, explain why.	Not Applicable	

Dual Use Research of Concern (DURC)	Information included in the manuscript?	In which section is the information available? (Reagents and Tools Table, Materials and Methods, Figures, Data Availability Section)
Could your study fall under dual use research restrictions? Please check biosecurity documents and list of **select agents and toxins** (CDC): https://www.selectagents.gov/sat/list.htm	Not Applicable	
If you used a select agent, is the security level of the lab appropriate and reported in the manuscript?	Not Applicable	
If a study is subject to dual use research of concern regulations, is the name of the **authority granting approval and reference number** for the regulatory approval provided in the manuscript?	Not Applicable	

Reporting

The MDAR framework recommends adoption of discipline-specific guidelines, established and endorsed through community initiatives. Journals have their own policy about requiring specific guidelines and recommendations to complement MDAR.

Adherence to community standards	Information included in the manuscript?	In which section is the information available? (Reagents and Tools Table, Materials and Methods, Figures, Data Availability Section)
State if relevant guidelines or checklists (e.g., **ICMJE, MIBBI, ARRIVE, PRISMA**) have been followed or provided.	Not Applicable	
For **tumor marker prognostic studies,** we recommend that you follow the **REMARK** reporting guidelines (see link list at top right). See author guidelines, under 'Reporting Guidelines'. Please confirm you have followed these guidelines.	Not Applicable	
For **phase II and III randomized controlled trials**, please refer to the **CONSORT** flow diagram (see link list at top right) and submit the CONSORT checklist (see link list at top right) with your submission. See author guidelines, under 'Reporting Guidelines'. Please confirm you have submitted this list.	Not Applicable	

Data Availability

Data availability	Information included in the manuscript?	In which section is the information available? (Reagents and Tools Table, Materials and Methods, Figures, Data Availability Section)
Have **primary datasets** been deposited according to the journal's guidelines (see 'Data Deposition' section) and the respective accession numbers provided in the Data Availability Section?	Yes	Data Availability
Were **human clinical and genomic datasets** deposited in a public access-controlled repository in accordance to ethical obligations to the patients and to the applicable consent agreement?	Not Applicable	
Are **computational models** that are central and integral to a study available without restrictions in a machine-readable form? Were the relevant accession numbers or links provided?	Yes	Data Availability
If publicly available data were reused, provide the respective **data citations in the reference list**.	Yes	References
